# Statistical significance of quantitative PCR

**DOI:** 10.1186/1471-2105-8-131

**Published:** 2007-04-20

**Authors:** Yann Karlen, Alan McNair, Sébastien Perseguers, Christian Mazza, Nicolas Mermod

**Affiliations:** 1 Institute of Biotechnology, University of Lausanne, 1015 Lausanne, Switzerland; 2 Max-Planck-Institute für Quantenoptik, 85748 Garching, Germany; 3 Department of Mathematics, University of Fribourg, CH-1700 Fribourg, Switzerland

## Abstract

**Background:**

PCR has the potential to detect and precisely quantify specific DNA sequences, but it is not yet often used as a fully quantitative method. A number of data collection and processing strategies have been described for the implementation of quantitative PCR. However, they can be experimentally cumbersome, their relative performances have not been evaluated systematically, and they often remain poorly validated statistically and/or experimentally. In this study, we evaluated the performance of known methods, and compared them with newly developed data processing strategies in terms of resolution, precision and robustness.

**Results:**

Our results indicate that simple methods that do not rely on the estimation of the efficiency of the PCR amplification may provide reproducible and sensitive data, but that they do not quantify DNA with precision. Other evaluated methods based on sigmoidal or exponential curve fitting were generally of both poor resolution and precision. A statistical analysis of the parameters that influence efficiency indicated that it depends mostly on the selected amplicon and to a lesser extent on the particular biological sample analyzed. Thus, we devised various strategies based on individual or averaged efficiency values, which were used to assess the regulated expression of several genes in response to a growth factor.

**Conclusion:**

Overall, qPCR data analysis methods differ significantly in their performance, and this analysis identifies methods that provide DNA quantification estimates of high precision, robustness and reliability. These methods allow reliable estimations of relative expression ratio of two-fold or higher, and our analysis provides an estimation of the number of biological samples that have to be analyzed to achieve a given precision.

## Background

Quantitative PCR is used widely to detect and quantify specific DNA sequences in scientific fields that range from fundamental biology to biotechnology and forensic sciences. For instance, microarray and other genomic approaches require fast and reliable validation of small differences in DNA amounts in biological samples with high throughput methods such as quantitative PCR. However, there is currently a gap between the analysis of the mathematical and statistical basis of quantitative PCR and its actual implementation by experimental laboratory users [1]. While qPCR has been the object of probabilistic mathematical modelling, these methods have not often been employed for the treatment of actual measurements. Therefore, the validity of the assumptions or simplifications on which these models are based is often unclear. At the other extreme, the treatment of laboratory measurements is often fairly empirical in nature, and the validity or reproducibility of the assay remains usually poorly characterized from an experimental and/or theoretical basis. Thus, practical qPCR methods usually do not allow mathematically validated measurements, nor the determination of the statistical degree of confidence of the derived conclusions. Consequently qPCR results have been questioned [2,3], with the consequence that semi-quantitative methods (e.g. end-point PCR) remain widely used.

**Figure 1:**
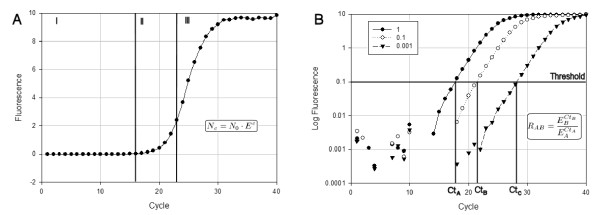
**Representations of real-time PCR amplification curves**. The three phases of the amplification reaction are shown either on a linear scale (panel A) or on a semi-log scale (panel B). Panel A represents a typical amplification curve, while panel B depicts amplification curves generated from serial dilutions of the same sample, either undiluted or diluted 10- or 1000-fold (indicated as 1, 0.1 or 0.001, respectively). During the lag phase (phase I), the fluorescence resulting from DNA amplification is undetectable above noise fluorescence in part A, while in part B, some data points take negative values and are not represented. This phase is used to evaluate the baseline "noise" of the PCR amplification. Exponential amplification of the DNA is detected in phase II (cycles 16 to 23, panel A). This phase of the amplification corresponds to the linear portion of the curve in panel B (closed circles). A threshold value is usually set by the user to cross the log linear portion of the curve, defining the threshold cycle value (*Ct*). Phase II is followed by a linear or plateau phase as reactants become exhausted (phase III). The inserted equations describe the dynamic of the amplification during phase II.

Quantitative PCR amplifications performed in the presence of a DNA-binding fluorescent dye are typically represented in the form of a plot as shown in Figure 1A, where the measured fluorescence is represented as a function of the PCR cycle number. An assumption that is common to all qPCR methods is that the fluorescence is directly correlated to the amount of double stranded DNA present in the amplification reaction [4]. The amplification curves are sigmoid shaped and can be split into three phases. Phase I (Figure 1A) represents the lag phase in which no amplification can be detected over the background fluorescence and statistical noise. This phase is used to evaluate the baseline fluorescent "noise". Phase II corresponds to the early cycles at which detectable fluorescence levels start to build up following an exponential behaviour described by the equation inserted in Figure 1A. On a log scale graph, this corresponds to the linear phase, illustrating the exponential dynamic of the PCR amplification (Figure 1B). During the later phase of the reaction, or phase III, the DNA concentration no longer increases exponentially and it finally reaches a plateau. This is classically attributed to the fact that one or more of the reactants become limiting or to the inhibition of amplification by the accumulation of the PCR product itself [5].

In a perfectly efficient PCR reaction, the amount or copy number of DNA molecules would double at each cycle but, due to a number of factors, this is rarely the case in experimental conditions. Therefore the PCR efficiency can range between 2, corresponding to the doubling of the DNA concentration at each cycle, to a value of 1, if no amplification occurs (Eq. 1 in methods). Furthermore, the efficiency of DNA amplification is not constant throughout the entire PCR reaction. The efficiency value cannot be measured during phase I, but it may be suboptimal during the first cycles because of the low concentration of the DNA template and/or sampling errors linked to the stochastic process by which the amplification enzymes may replicate only part of the available DNA molecules [6]. Quantitative PCR is used under the assumption that these stochastic processes are the same for all amplifications, which may be statistically correct for *N*_0 _values that are large enough so that sampling errors become negligible [7]. The efficiency reaches a more or less constant and maximal value that may approach 2 in the exponential amplification of phase II, and it finally drops to a value of 1 during phase III. This implies that any appropriate analytical method should focus on phase II of the amplification where the amplification kinetic is exponential. Therefore, the first step in any qPCR analysis is the identification of phase II, which is more conveniently performed when data are represented on a log scale (Figure 1B).

Another assumption of qPCR is that the quantity of PCR product in the exponential phase is proportional to the initial amount of target DNA. This is exploited by choosing arbitrarily a fluorescence threshold with the condition that it lies within the exponential phase of the reaction. When fluorescence crosses this value, the cycle is termed the "Threshold cycle" (*Ct*) or "Crossing Point", and the higher the *Ct*, the smaller the initial amount of DNA. This is illustrated in Figure 1B, which displays qPCR amplifications performed on serial dilutions of a cDNA sample.

One of the first and simple methods to process qPCR data remains a set of calculations based solely on *Ct *values and is currently known as the Δ*C*_*t *_method [8,9]. However, as such, this method assumes that all amplification efficiencies are equal to 2 or at least equal between all reactions. Therefore it does not take into consideration possible variations of amplification efficiencies from one sequence or sample to the other. Thus, the Δ*C*_*t *_method may not accurately estimate relative DNA amounts from one condition or one sequence to the other. Consequently, other methods of data processing have been developed to estimate the efficiency of individual PCR amplifications [10-13]. Alternatively, amplification curves can be directly fitted with sigmoid [14] or exponential functions (Methods section, Eq. 6 and Eq. 8) in order to derive the original amount of template DNA (Eq. 7 and Eq. 9).

**Figure 2:**
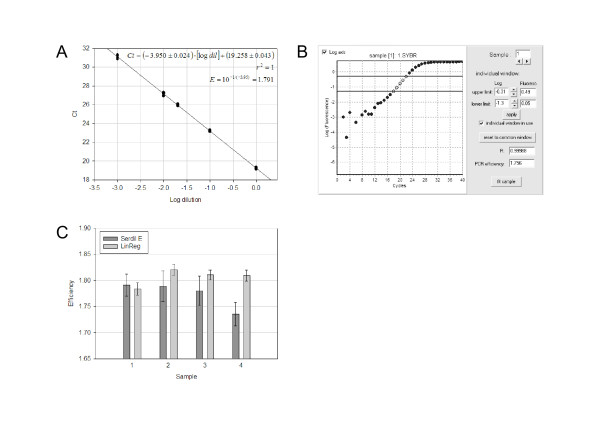
**Measurement of the efficiency of a PCR reaction**. A: Estimation of the efficiency using the Serial dilution (SerDil) method. Five dilutions of a cDNA sample were amplified using the fibronectin (FN) amplicon. Each dilution was analyzed with five replicates PCR reactions and each data point represents one *Ct *value determined as in Figure 1B. Linear regression parameters and calculation of the efficiency value are shown in the inserted textbox. B: Screenshot of the LinReg PCR program analysis window, which allows the estimation of the efficiency value from each set of amplification curves [13]. Data correspond to one of the reactions performed from the undiluted sample used in part A. C: Comparison of the efficiency values obtained using the Serial dilution and LinReg methods for the FN amplicon. Efficiency values were determined from four independent cDNA samples using the Serial Dilution method as in part A, or the LinReg method as in part B. For each sample, efficiency value were either determined from one linear regression performed on 24 reactions altogether (Serdil) and error bars calculated from the standard deviation on the slope as determined from the linear regression method or the individual efficiency values determined from each of the same 24 PCR reactions (LinReg) were averaged, and error bars represent the standard deviation on the set of values.

Methods to estimate amplification efficiency can be grouped in two approaches, both of which rely on the log-linearization of the amplification plot. The most commonly used method requires generating serial dilutions of a given sample and performing multiple PCR reactions on each dilution [10,12]. The *Ct *values are then plotted versus the log of the dilution (Figure 2A) and a linear regression is performed (Eq. 4) from which the mean efficiency can be derived (Eq. 5). As stated above, this approach is only valid if the *Ct *values are measured from the exponential phase of the PCR reaction and if the efficiency is identical between amplifications.

The other method currently used to measure efficiency is based on Eq. 3, which associates an efficiency value with each PCR reaction [12]. This approach has been automated in different programs [15], one of which, termed LinReg PCR [13], was used in this study. LinReg identify the exponential phase of the reaction by plotting the fluorescence on a log scale (Figure 2B). Then a linear regression is performed, leading to the estimation of the efficiency of each PCR reaction.

None of the current qPCR data treatment methods is in fact fully assumption-free, and their statistical reliability are often poorly characterized. In this study, we evaluated whether known mathematical treatment methods may estimate the amount of DNA in biological samples with precision and reliability. This led to the development of new mathematical data treatment methods, which were also evaluated. Finally, experimental measurements were subjected to a statistical analysis, in order to determine the size of the data set required to achieve significant conclusions. Overall, our results indicate that current qPCR data analysis methods are often unreliable and/or unprecise. This analysis identifies novels strategies that provide DNA quantification estimates of high precision, robustness and reliability.

## Results

Quantitative PCR usually relies on the comparison of distinct samples, for instance the comparison of a biological sample with a standard curve of known initial concentration, when absolute quantification is required [16], or the comparison of the expression of a gene to an internal standard when relative expression is needed. The equation inserted in Figure 1B is used to calculate the ratio of initial target DNA of both samples (Eq. 2). The error on the normalized ratio depends on the error on the *Ct *and the error on the efficiency, and it can be estimated from Eq. 11. However, the range and relative importance of the various components, and the origin of the error on practical measurements remain poorly characterized.

To evaluate the reproducibility of *Ct *measurements and their associated error, we generated a set of 144 PCR reaction conditions corresponding to various target DNA, cDNA samples and dilutions (see Additional file 1 for a description of targeted genes and amplicons). Each of these 144 reaction conditions was replicated by performing 4 or 5 independent PCR amplifications. This yielded a complete dataset of 704 amplification reactions which collection of raw data is given in additional file 2. Individual *Ct *values corresponding to each reaction conditions were averaged, providing a set of 144 *Ct *values and their associated errors. The standard deviation (SD) shows an increase of the error with higher *Ct *values, with SD values smaller than 0.2 for *Ct *up to 30 cycles, and spreading over 0.8 for *Ct *higher than 30 (Additional File 3). Thus, all replicates with SD above 0.4 were excluded, which corresponds to some of the reactions with *Ct *above 30 in this study. We conclude that *Ct *between 15 and 30 can be reproducibly measured leading to a dynamic range of 10^5^, which is within the 4 to 8 logs dynamic range reported in other studies [17]. In these conditions, *Ct *value determination is unlikely to be a major source of error when calculating normalized ratio of expression. Thus, we then focused on the estimation of efficiency.

### Estimation of the efficiency of a PCR reaction

We compared estimates of the efficiency obtained from two distinct methods: the generally used serial dilution (Figure 2A) and the alternative LinReg method (Figure 2B). With our experimental setup, estimation of the efficiency with the serial dilution method requires a set of 24 PCR reactions for a given sample and a given amplicon, using serially diluted template DNA. The efficiency obtained was compared to the average efficiency estimated from each of the reactions with the LinReg method. Efficiency estimates are comparable when looking at values given in Figure 2A and 2B, but they differed when comparing the efficiencies obtained from one of the four DNA samples (Figure 2C). Thus, we questioned whether the two methods provide statistically similar measures of efficiency, and whether they display similar reproducibility.

The statistical equivalence of the LinReg and serial dilution methods was assessed using an analysis of variance (ANOVA, Table 1), which indicated that the efficiency averages are not significantly different between the two methods except for one amplicon, corresponding to the Connective Tissue Growth Factor (CTGF) cDNA (*p *< 0.05). This may be linked to the fact that this gene is expressed at very low levels and because of the reduced size of the data set, as some data had to be discarded because signal was undetectable (*Ct *≥ 40). Also, some estimates were taken from PCR displaying Ct values in the 35 – 40 range. Thus, statistically significant difference between the two methods may likely result from the smaller dataset and/or the use of reactions with *Ct *values outside of the optimal range. The reproducibility of the LinReg method appears to be overall higher than that of the serial dilution method (Figure 2C). An F-test performed over the averaged variance of each method indicated that for each set of primer, the difference between the variance of the serial dilution and LinReg methods is very significant, with *p*-values well below 0.001 (Table 1).

**Table 1:** Comparison between serial dilution and LinReg for the measurement of efficiency

Amplicon	Test of equality*	Test of variance**
Cav	p = 0.24	p < 0.001
CTGF	p < 0.001	p < 0.001
Eln	p = 1	p < 0.001
FN	p = 0.65	p < 0.001
L27	p = 0.8	p < 0.001
Perl	p = 1	p < 0.001
PAI-1	p = 0.93	p < 0.001

* Single one-way ANOVA was applied to the complete set of data to assess whether both the Serial dilution and the LinReg methods give similar averaged efficiencies (*H*_0_: efficiencies are equal, *H*_1_: efficiencies are different): a p-value below 0.05 indicates that measured efficiencies are significantly different. **F-test assessing whether the variance (error) induced by each method is the same [*H*_0_: variances are equal, *H*_1_: *var*(serial dilution) > *var*(LinReg)]: p < 0.001 indicates that the variance obtained with the serial dilution is significantly greater than that of LinReg.

Overall, we conclude that the two methods display comparable accuracy in measuring efficiency values of a set of reactions. Statistically, this implies that these methods provide acceptable estimator of the efficiency. However, LinReg appears to be more robust, as lower variances were obtained. Furthermore, LinReg can be mathematically justified when the PCR amplification is in the exponential phase (see Additional File 4).

### Experimental parameters influencing efficiency determination

**Figure 3:**
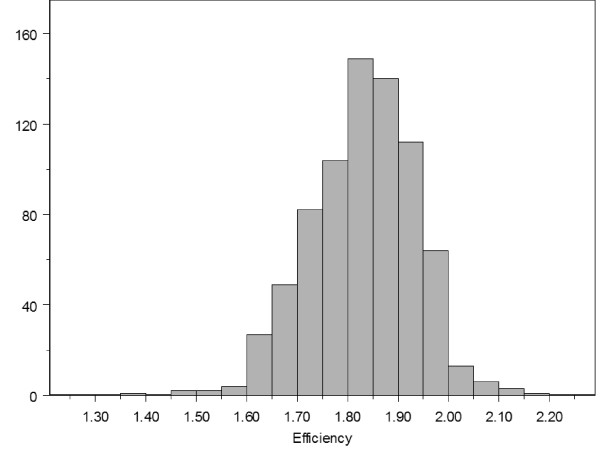
**Distribution of efficiency values in the complete data set**. Efficiency of 704 PCR reactions encompassing different cDNA samples, primers and dilutions are shown as determined using the LinReg method, as done in Figure 2B.

Next, we wished to determine which of the experimental variables may affect the precision of the estimation of efficiency. This was evaluated on the complete set of quantitative PCR reactions. Figure 3 shows the distribution of the efficiencies measured for all reactions. Efficiencies ranged from 1.4 to 2.15 with a peak value around 1.85. Theoretically, efficiencies can only take values between 1 and 2, and therefore they are expected to deviate from a normal distribution, as indicated by a Kolmogorov-Smirnoff test (not shown). However, the distribution appears to be sufficiently symmetrical to be considered normal, such that classical statistical tests can be validly performed.

First, we determined if single PCR parameters (amplicons, cDNA samples, *Ct *value, etc.) may influence the efficiency value by performing a multiple ANOVA test on all values. The first four entries of Table 2 indicate that the efficiency is most dependent upon the amplicon and relatively less on cDNA samples, as indicated by high F values, both of these effects being highly significant (*p *< 0.001). However, efficiency was not found to depend on the *Ct *nor the dilution, showing that efficiency is solely dependent on the kinetic of the PCR reaction in the exponential phase and not on the initial condition (i.e. the amount of initial template). Possible interactions between pairs of parameters that affect the efficiency (co-dependence) were also assessed, showing that amplicon-dependent effects on the efficiency are modulated by the type of sample. Interestingly, while the dilution is not significantly influencing the efficiency, it can significantly modulate the effect of the primers and the samples. This is consistent with the presence of inhibitor(s) in samples that would affect PCR reactions at the highest concentrations. For instance, salts or competing genomic DNA would be expected inhibit the interaction of primers and target DNAs differently. None of the other co-dependences were of any significance. Since the *Ct *and dilution are not independent parameters, two ANOVA test were run excluding either one. The *Ct *did not have a significant effect on the efficiency even when the dilution parameter was not taken into account. On the other hand, the dilution effect was not affecting efficiency significantly by itself but it modulated the effect of the amplicon. Either way, these results indicate clearly that the *Ct *has no direct effect on the efficiency.

**Table 2:** Parameters influencing the efficiency of qPCR reactions.

Parameter	Df	F-value	p-value
Amplicon	6	219.331	<0.001
Sample	10	7.227	<0.001
*Ct*	1	0.646	0.422
Dilution	4	2.111	0.078
Amplicon:Sample	15	4.525	<0.001
Amplicon:Ct	6	1.957	0.07
Amplicon:Dilution	20	2.576	<0.001
Sample:Ct	10	1.226	0.271
Sample:Dilution	37	1.512	0.029
*Ct*: Dilution	4	1.872	0.114
Residuals	700		

A multiple ANOVA test was performed on efficiencies obtained from 700 reactions. Dependence with amplicon sequence, sample, Ct and dilutions were tested as well as all combinations of co-dependence. Df indicates the degree of freedom. The larger the F-value, or the smaller the p value, the more significant is the effect of the corresponding parameter.

Overall, these results indicate that efficiencies are highly variable among PCR reactions and that the main factor that defines the efficiency of a reaction is the amplicon. This is consistent with the empirical knowledge that primer sequences must be carefully designed in quantitative PCR to avoid non-productive hybridization events that decrease efficiency, such as primer-dimers or non-specific hybridizations. Efficiency might also depend upon the dilution for a minority of the cDNA samples, indicating that dilute samples should be preferred to obtain reliable efficiency values.

### DNA quantification models

The models we evaluated in this study can fall into two different groups: being derived from either linear or from non-linear fitting methods. Comparison of qPCR data using models based on non-linear fitting methods (Eq. 6 and Eq. 8) is done simply by calculating the ratio of the initial amount of target DNA of each amplicon (Eq. 7 and Eq. 9) as in the first part of Eq. 2. The standard deviation of the ratio on a pool of replicate is calculated using Eq. 10. Note that in this case, errors resulting from the non-linear fitting itself are not considered in the analysis.

Linear fitting methods also allow the estimation of the initial level of fluorescence induced by the target DNA. For instance, Eq. 3, upon which the LinReg method relies to determine efficiency, can also be used to determine *F*_0 _as the intercept to the origin of a linear regression of the log of fluorescence. This figure can then be used to calculate relative DNA levels (Eq. 2). This calculation method was termed *LRN*_0_.

However, even small errors on the determination of the efficiency will lead to a great dispersion of *N*_0 _values due to the exponential nature of PCR (Eq. 2). Therefore, we considered alternative calculation strategies, whereby the efficiency is averaged over several reactions rather than using individual values, which should provide more robust and statistically more coherent estimations. We therefore evaluated the use of efficiency values calculated in three different manners.

As the amplicon sequence is the main contributor to the efficiency, we used the efficiency averaged over all cDNA samples, dilutions and replicates of a given amplicon, as a more accurate estimator of the real efficiency than individual values. The error on the efficiency is no longer considered in the calculations of relative DNA concentrations, thus assuming that the estimator is sufficiently precise so that errors become negligible. This model is termed below (*PavrgE*)^*Ct*^.

Alternatively, the small influence of the sample upon the efficiency was taken into account by averaging the efficiencies obtained for each dilutions and replicates of a given cDNA sample and a given amplicon. Thus, for a given cDNA sample and amplicon, one efficiency value is obtained from 24 PCR reactions. This value is used in further calculations, assuming again the average value to be a sufficiently good estimator of the efficiency so that the relative error may not be taken into account. This model was named (*SavrgE*)^*Ct*^.

Finally, we tested a model in which the efficiency is estimated individually for each set of replicated reactions. This was addressed by averaging the efficiency of each replicates of a given amplicon, cDNA sample and dilution. This model is referred below as *E*^*ct*^. These three models are summarized in Table 3.

**Table 3:** Models for the use of single reaction efficiencies

	Grouping of individual efficiencies for average determination				
		
Model	Amplicon (7)	cDNA (4)	Dilutions (5)	Replicates (5)	Reactions (700)
(*PavrgE*)^Ct ^*	individual	pooled	pooled	pooled	100
(*SavrgE*)^*Ct*^	individual	individual	pooled	pooled	25
*E*^*ct*^	individual	individual	individual	pooled	5

*Each line indicates how single efficiency values are grouped in each of the calculation models. For instance, the (*PavrgE*)^*Ct *^model line indicates that one individual amplicon is chosen out of the 7 available ones, then efficiencies estimated from the different cDNA samples, dilutions and replicates are all pooled, leading to the determination of one efficiency value from 100 reactions. In the (*SavrgE*)^*Ct *^model, individual efficiency values are calculated for each cDNA sample and each amplicon averaging efficiency values from the 5 replicate reactions performed on the 5 dilutions (25 values overall).

### Evaluation of the quantitative PCR calculation models

**Figure 4:**
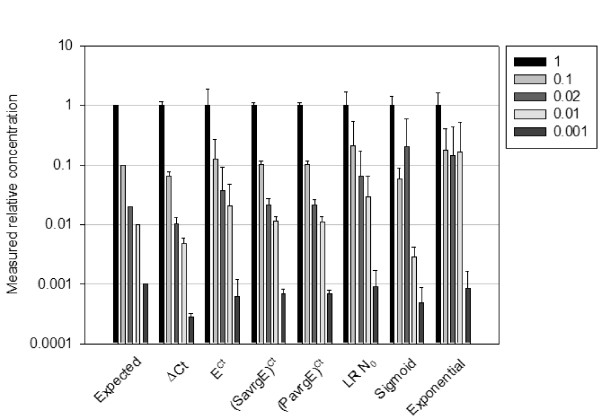
**Comparison of the different calculation models when applied to samples of known relative concentrations**. Each cDNA samples serial dilutions were processed with the indicated models, measured concentration were expressed as relative to the undiluted sample. Then results of all amplicons were averaged for a given model.

The dilutions of a given sample form a coherent set of data, with known concentration relationships between each dilution. Each calculation model was therefore used on each dilution series, using the undiluted sample for normalization. All data can be presented as measured relative concentrations, the undiluted dilution taking the relative concentration value of 1, the 10-fold dilution taking the value of 0.1, the 50-fold dilution a value of 0.02, and so on. The measured relative concentrations for all dilutions, samples and primers and the associated errors were calculated using each model from the complete dataset of 704 reactions. For the models giving a direct insight to the initial *N*_0 _values, *N*_0 _were averaged for each amplicon and cDNA sample, and they were plotted in comparison with the expected concentrations relative to the undiluted samples (Figure 4). The models were evaluated on three criteria: resolution, precision and robustness.

We defined the resolution as the ability of a model to discriminate between two dilutions. Relative concentrations were compared pair-wise between adjacent dilutions. Typically, it can be seen in Figure 4 that models did not give uniformly coherent results. For instance, models that do not rely on explicit efficiency values, such as the sigmoid or exponential models, are unable to discriminate between the 0.1 and 0.02 relative concentrations, which shows a lack of resolution in this range of dilutions. The Δ*Ct*, (*PavrgE*)^*Ct *^and (*SavrgE*)^*Ct *^models performed well under this criterion, allowing easy discrimination of the 10-fold and 50-fold dilutions in this example.

The resolution was statistically evaluated with a coupled ANOVA-LSD t-test, which is a two step analysis of variance (ANOVA) coupled to a t-test run under the *Least Significant Difference method (LSD) *[18]. Unsurprisingly, the ANOVA test indicated that for all models at least one of the measured concentrations differed significantly from the others as expected (data not shown). To further assess if all measured concentrations significantly differ from one another, or if some are undistinguishable, a coupled t-test was performed on pairs of adjacent dilutions in a given serial dilution series. Results are summarized in Table 4. All models were able to discriminate the undiluted condition from the 10-fold dilution (highly significant, p < 0.01). The sigmoid and exponential models did not discriminate further dilutions. The Δ*Ct*, *E*^*Ct *^and LR*N*_0 _p-value indicate that these models could discriminate the 10-fold from the 50-fold dilution, but not further dilutions. The (*SavrgE*)^*Ct *^and (*PavrgE*)^*Ct *^models were able to discriminate the 10-fold from the 50-fold dilution and were at the limit of significance when comparing the 50-fold with the 100 fold dilutions (significant, p < 0.1). Finally, none of the models were able to discriminate the 100 fold from the 1000 fold dilution. These comparisons indicated that (*SavrgE*)^*Ct *^and (*PavrgE*)^*Ct *^models performed equally well in this assay, followed by Δ*Ct*, *E*^*Ct *^and LR*N*_0_, while the sigmoid or exponential models were of low resolution. These results also illustrate that more dilute samples are generally more difficult to discriminate, as expected from the finding that variance increases with higher *Ct *values (Additional File 3).

**Table 4:** Resolution of each calculation model

Relative concentrations	Δ*CT*		E^Ct^		(Savrg E)^Ct^		(Pavrg E)^Ct^		LR N_0_		Sigmoid		Exponential	
							
	t-value	p-value	t-value	p-value	t-value	p-value	t-value	p-value	t-value	p-value	t-value	p-value	t-value	p-value
1 – 0.1	122.7	0.000	18.6	0.000	141.7	0.000	141.3	0.000	18.9	0.000	29.1	0.000	18.2	0.000
0.1 – 0.02	7.3	0.000	1.9	0.030	12.6	0.000	12.6	0.000	3.5	0.000	-4.4	N/A	0.8	0.225
0.02 – 0.01	0.7	0.232	0.4	0.360	1.6	0.052	1.6	0.052	0.9	0.190	6.1	0.000	-0.4	N/A
0.01 – 0.001	0.5	0.325	0.3	0.375	1.3	0.105	1.2	0.107	0.5	0.306	0.1	0.478	2.7	0.004

A ANOVA coupled t-test was performed to determine which dilutions are statistically different from one another. The comparison of adjacent dilutions are shown. A p-value higher than 0.05 indicate that the model is unable to discriminate between the two adjacent dilutions. N/A stands for not available and indicate that the expected higher concentration of the comparison was in fact calculated to be lower from the qPCR results.

The precision of a model is defined by its ability to provide expected relative concentrations of the known dilutions. Again Figure 4 shows that the (*PavrgE*)^*Ct *^and (*SavrgE*)^*Ct *^models provide precise relative concentration values over all dilutions, with the measured relative concentrations matching the expected ones. Estimations obtained by the Δ*Ct *model appear to be less reliable, with a systematic under-representation of concentrations. This result is expected since all of our amplicons have efficiencies that are below 2 (see Additional File 5).

We statistically evaluated the precision of each model by plotting the expected relative concentration against the measured relative concentration averaged from all primers and samples (Additional File 3). A linear regression was done on the data obtained from each model and a t-test was performed to determine if the slope is statistically different from 1. A low p-value in Table 5 is associated to a high probability that the slope is different from 1, indicative of a poor correlation between expected and measured values. As before, the (*PavrgE*)^*Ct *^and (*SavrgE*)^*Ct *^models outperformed all other models, being more precise than the and sigmoid models, the exponential, Δ*Ct *and LR *N*_0 _displaying lowest precision.

**Table 5:** Precision of each calculation model

Model	Slope	SD	p-value	r^2^
Sigmoid	0.085	1.225	4.4E-12	0.00004
LR N_0_	1.830	0.621	4.7E-26	0.07441
Exponential	0.707	1.047	0.004	0.00421
E^Ct^	1.103	0.127	1.2E-13	0.40965
ΔCt	0.689	0.013	1.4E-149	0.96041
(Pavrg E)^Ct^	0.994	0.026	0.021	0.93213
(Savrg E)^Ct^	0.996	0.027	0.170	0.92419

Expected and measured relative concentrations were plotted and a linear regression was performed (Additional File 3). The slopes values were submitted to a t-test to evaluate if they are statistically different from 1, which would indicate a poor correlation between expected and measured concentrations. A p-values smaller than 0.05 indicate that the slope is significantly different from 1. Correlation coefficient (r^2^) values indicate the width of the spreading of individual data around the linear regression line. Higher r^2 ^indicate more robust models.

Finally, the robustness is related to the variability of the results obtained from a given model, and it indicates whether trustable results may be obtained from a small collection of data. For instance, a model could be very precise (eg providing a slope of 1) with a large data set, but the distribution of the points around the regression line could be very dispersed. Such a model would not be robust as a small data set would not allow precise measurements. Thus, the robustness of a model was estimated from the standard deviation of the slope and the related correlation coefficient of the linear regression (*r*^2^), with higher *r*^2 ^values indicating more robust models. Three models showed high robustness, the Δ*Ct*, (*PavrgE*)^*Ct *^and (*SavrgE*)^*Ct*^, followed by *E*^*Ct *^(Table 5). Overall, only two calculation models combine high resolution, precision and robustness, namely the (*PavrgE*)^*Ct *^and the (*SavrgE*)^*Ct *^methods. However, only the slope of the (*SavrgE*)^*Ct *^did not statistically differ from 1.

### Model evaluation on a biological assay of gene expression regulation

Usually, experimenters are interested in the difference between two conditions (with versus without a drug, sane versus metastatic tissue, etc...) [19-21], for instance to determine whether the expression of the gene of interest is induced or repressed upon treatment or between samples. So the useful figure is the normalized induction ratio (Eq. 13). We set up to use the most promising approaches on samples of biological interest. NIH-3T3 fibroblastic cells were incubated with the TGF-β growth factor for 4 hours, as it is known to induce the expression of a number of extracellular matrix protein genes. For this experiment, the CTGF, FN and PAI-1 genes were chosen, for they were shown to be induced at various levels by the growth factor in fibroblasts [22,23]. The total mRNA of three independent biological samples from the induced as well as the non-induced condition were mixed and processed as before. The expression levels of these genes were normalized to the ribosomal L27 protein gene expression used as an invariant mRNA, so as to correct for differences in mRNA recovery or reverse transcription yield. Following the results of the previous section, only the (*SavrgE*)^*Ct*^, (*PavrgE*)^*Ct *^and Δ*Ct *methods were used.

**Figure 5:**
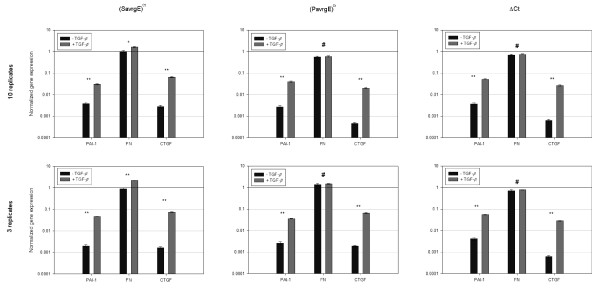
**Comparison of the (*SavrgE*)^*Ct*^, (*PavrgE*)^*Ct *^and ΔCt methods to quantify gene expression regulation**. cDNAs were prepared from RNA extracted from fibroblastic cells induced or not by TGF-β treatment, as described in the Materials and methods. Expression as determined from the mRNA levels of the plasminogen activator inhibitor 1 (PAI-1), fibronectin (FN) and connective tissue growth factor (CTGF) genes were normalized to those of the ribosomal L27 protein, used as an invariant internal reference. Normalized gene expression was calculated using the (*SavrgE*)^*Ct*^, (*PavrgE*)^*Ct *^or the ΔCt methods, as indicated, using either the complete set of 10 replicate assays (top histograms), or using just three measurements (first three assays of the series, bottom histograms). Error bars represent standard deviations on the normalized ratio. A t-test was performed on the normalized gene expression to check whether the expression were statistically different between the induced and the non-induced state (* = p < 0.05, ** = p < 0.001, # = p > 0.1).

Ten replicate PCR reactions were performed for each condition (induced or non-induced) and normalized expression values obtained with (*SavrgE*)^*Ct *^(*SavrgE*)^*Ct*^, (*PavrgE*)^*Ct *^or Δ*Ct *are shown in Figure 5. Fibronectin is expressed at high levels but it is only moderately induced by the growth factor (Additional File 1), while PAI-1 and CTGF have much lower expression levels but higher induction ratios (Figure 5, top panels). The three methods yielded consistent results overall. However, the low induction ratio of the fibronectin gene was statistically significant with (*SavrgE*)^*Ct *^(p < 0.05) but not with (*PavrgE*)^*Ct *^(0.38) or Δ*Ct *(p = 0.39).

To assess whether the relative performance of the three models depends critically on the number of replicate assays, the analysis was repeated, but taking into account only the first three values obtained from the set of 10 replicates. Similar results were obtained (Figure 5, bottom panels), and the small induction of the expression of the FN gene was again only detected using the (*SavrgE*)^*Ct *^model. Thus, small differences in gene expression are also more reliably estimated from this model with a low number of replicates commensurate with usual experimental procedures.

### Dataset size required to achieve statistical significance

In the above example, independent biological samples were mixed so as to decrease the variability associated with cell culture and mRNA isolation. Therefore, this study provides the statistical significance that may be expected just from the intra-assay variability in the qPCR process. However, statistical significance will also depend on the inter-assay, or biological variability. To assess the statistical significance associated to particular conclusions on gene expression regulation, replicates of induction experiments are usually generated and, in most experimental studies, the number of biological replicates is low, being typically obtained from 3–6 independent biological samples.

Thus, we wished to determine how many biological replicates may be necessary to obtain statistically reliable results, depending upon the variability of the assay (Eq. 15). Using the data from the 10 replicates to estimate the intra-assay variability, we found that the standard deviation is proportional to the induction ratio value (Additional File 1). This is shown by coefficient of variation (CV) values being conserved for all induction ratios at a level just below 15%, irrespective of the calculation method. Use the set of three replicate assays resulted in more variable but comparable CV values around or lower than 15%, which is in agreement with other published data [17]. However, inter-experiment biological variability will be specific to each experimental system. The true variability of the PCR assay (intra-assay and biological inter-assay variability) is higher, typically with overall CV values ranging around 30% to 50% (own unpublished results and [24]). Another parameter influencing the number of replicates needed to assess statistical reliability is the domain (range) of confidence of the measure. This value is defined as the largest acceptable error on the measure and it is set arbitrarily by the experimenter. Thus, setting a domain of 20% indicates that the estimated induction should fall within 20% of the real value. It follows that the larger the domain of confidence, the lower the number of replicates needed.

Table 6 provides the number of independent measurements that is required to achieve a statistically significant measurement from any given induction ratio. Taking the minimal theoretical CV of 15%, obtaining an induction ratio within a domain of 10% of the real value would require 9 independent induction measurements. If one accepts a range of 30%, only one value is expected to be required. However, considering the biological variation in a CV value of 50%, and setting a 10% range of confidence, then 97 independent measurements would be needed to achieve statistically valid conclusions, which is unpractical in most cases. One therefore needs to accept a range of confidence of 50% to pull this value down to a feasible experimental set of 4 independent biological samples. Thus, with such settings, qPCR may be reasonably used to detect induction ratio around 2-fold or higher.

**Table 6:** Number of measurement replicates needed to reach statistical significance

	Range (%)				
	
CV value (%)	10	20	30	40	50
15	9	3	1	1	1
30	35	9	4	3	2
40	62	16	7	4	3
50	97	25	11	7	4

The number of replicates needed to reach statistical significance depends on the reproducibility of the measures (CV) and the range of confidence one wants to achieve (the real value being within the indicated percentile from the estimator qPCR value). The confidence level α was set to 0.05 (*z*_0.975 _= 1.960).

## Discussion

The simplicity of producing quantitative PCR data has overshadowed the difficulty of making a proper analysis of those data. Although the principle of qPCR is theoretically well described, analysis of the experimental data can become very difficult if one is not aware of the different assumptions that the different models are based on, and of their resulting limitations. Furthermore, a systematic evaluation of the relative performance of the models used for the treatment of experimental measurements and a description of their statistics are currently lacking. Thus, no single method has gained a general acceptance in the community of experimentalists.

In this study, we reviewed the mathematical basis and assumptions of previously described calculation methods and evaluated their ability to provide quantitative results from a practical dataset size. Initially, we first evaluated previously reported methods and concluded that an estimation of the PCR amplification efficiency is prerequisite to obtaining precise quantification, in agreement with other studies [6,25-28]. However, we found that the error associated with the determination of the efficiency value may render measurements of little statistical significance. Therefore, in addition to evaluating previously proposed general data processing strategies (Δ*Ct*, *E*^*Ct*^), we generated new methods or variations (exponential fit, (*PavrgE*)^*Ct *^and (*SavrgE*)^*Ct*^, *LRN*_0_), and we compared all approaches with datasets generated from several independent genes and biological conditions.

### Estimation of the PCR efficiency

The classical serial dilution and the newer LinReg methods used for measuring efficiency were both found to provide good estimator values of the efficiency, as based on an ANOVA analysis. However, the efficiency values were not uniformly equivalent when comparing both methods, as they were significantly different for some of the assay genes. The larger variability of efficiency values obtained with the classical serial dilution method with all test genes led us to conclude that it was not an estimator as accurate as LinReg. In addition, while both methods are very sensitive to changes of the concentration of potential inhibitors present in the sample upon serial dilutions, the serial dilution method does not allow the assessment of such effect while LinReg does [13]. Furthermore, LinReg requires much less PCR reactions to determine efficiency and is faster to implement. Results presented here show that consistent efficiency estimates can be obtained for a variety of target genes with this method.

### Factors affecting the efficiency value

The large variation associated with efficiency estimations, even from duplicate analysis of the same sample, led us to analyse the determinants of this variability. This analysis showed that efficiency is strongly dependent on the primer sequence. These results are in accordance with the common knowledge that careful design of the PCR primers is required to obtain useable PCR amplifications data and high efficiency values [29,30]. Dependence of the efficiency on the primer sequences may be explained by interfering reactions that would decrease PCR efficiency depending on the primer pair, such as formation of primer dimers, intra-strand hybridization or unspecific hybridization to other cDNA sequences.

Assay of independent biological samples was also found to significantly affect the efficiency, but to a lesser extent. Others have observed that sample to sample variations may predominate, which may reflect differences in sample preparation methods and/or distinct biological systems [31]. These effects can be related to cell-specific contaminants and/or to exogenous contaminants introduced during sample preparation that may interfere with the assay [29,30]. Indeed, use of undiluted reverse transcriptase reaction samples was found to decrease efficiency values significantly and to increase variability between samples (YK, unpublished results). However, such effects should be alleviated in dilute samples such as those used here, and we found that the average efficiency value does not correlate significantly with the dilution factor, at least for the dilution range used in this study. This indicates that in the conditions used, the variation associated with the samples does not result primarily from chemical contaminants that would interfere directly with the DNA elongation reactions. Interestingly, the efficiency values are not dependent on the measured *Ct*, which reflects both the initial DNA concentration in the extract and the dilution ratio, further strengthening the conclusion that the concentration of impurities in the sample or the initial *N*_0 _concentration are not the main determinants of the PCR efficiency. Therefore, the distinct efficiencies obtained from independent samples should also reflect other properties of the sample that are not affected by dilution.

For instance, the presence of damaged or nicked cDNA in the sample has been shown to affect PCR efficiency [32]. This may result in the linear amplification of shorter DNA fragments, as opposed to the exponential amplification of correct length DNA from the undamaged cDNA, and in a decrease of available nucleotides around the Ct cycle affecting the observed efficiency. In addition, if linearly amplified truncated DNA strands still contribute a significant proportion of fluorescence at the *Ct*, increase in fluorescence would reflect both the efficiency of the amplification of DNA template of correct length (exponential amplification) and a lower efficiency value corresponding to the amplification of shorter molecules (linear amplification). Similarly, the presence of incomplete elongated cDNAs, base hydrolysis or chemical oxidation also impairs polymerase progression, leading to the unidirectional amplification of shorter products that could also decrease PCR efficiency [33]. Variations in the ratio of non-functional to functional templates would thus explain changes in the apparent amplification efficiency from one sample to the next irrespective of the dilution.

### Evaluation of various qPCR calculation models

Models were evaluated under 3 criteria: resolution, precision and robustness. Resolution is a measure of the ability of a model to discriminate two successive dilutions. Precision is the correlation between measured and expected concentrations. Finally the robustness is a measure of the dispersion of the measured values around the expected concentrations.

Overall, two calculation models stand out: the (*PavrgE*)^*Ct *^and the (*SavrgE*)^*Ct *^models, as these are among the top scoring methods on the three evaluation criteria. However, (*PavrgE*)^*Ct *^shows a small but statistically significant bias when comparing the obtained and expected values, suggesting that it slightly underestimates the more dilute DNA concentrations. In contrast, results calculated from (*SavrgE*)^*Ct *^cannot be statistically distinguished from the expected data and are thus of higher precision. In addition, (*SavrgE*)^*Ct *^displayed a higher resolution than (*PavrgE*)^*Ct *^when assessed on biological samples. These models are followed by *E*^*Ct*^, which is of lower but consistent resolution, robustness and precision.

The Δ*Ct *model stands apart from all other models. Firstly, because it is the first model ever having being used in automated quantitative PCR, but also because of its properties as analyzed here. As expected, this model very significantly underestimates actual DNA concentrations, with a clear statistical indication that it is of low precision. However, it mediates the highest robustness value of all methods. Thus, this model is quite unprecise, but it yields very reproducible results. The Δ*Ct *model may therefore be of interest for screening purposes, as its strong robustness and ease of use makes it ideal to analyze large collections of biological samples with few replicates, for instance to screen for changes in the expression a large number of genes, after which a finer analysis on the genes displaying interesting expression profiles may be performed using the (*SavrgE*)^*Ct *^or (*PavrgE*)^*Ct *^models. It must be emphasized here that if PCR is performed with carefully designed and optimized primers that yield high efficiency [34], then the Δ*Ct *model would be the best model of all. Unfortunately obtaining such primers is labour-intensive and costly, when not impossible.

Finally the sigmoid, exponential and LR *N*_0 _models analysed here are least suitable for quantitative PCR analysis as they have a low resolution and/or precision, and because they display very low robustness. Improved versions of the original sigmoid model [14] used here has recently been reported [35,36], which should result in increased robustness. In parallel to the sigmoid fitting methods analyzed here, we also evaluated several other sigmoid fitting algorithms, which performances were either similar or even less accurate than the method used here (unpublished data). This observation is in accordance with Feller's conclusions that different S-shaped curves can be similarly fitted with various sigmoid models [37], each providing distinct *N*_0 _value from its own set of parameters. Thus sigmoid fit methods such as the logistic model used above are purely descriptive, and biological conclusions drawn from the fitting parameters may be unreliable.

Perhaps surprisingly, the exponential fitting method also scored with very low performance, despite the expected exponential nature of DNA amplification by PCR. This may result in part from poorly characterized borders of the exponential phase, leading to the fitting of experimental points that are already in phase III. Alternatively it may result from the possible non-exponential nature of PCR that would result from both linear and exponential amplification, as discussed above. The exponential and sigmoid methods are based on descriptive models. They often produce outlier *N*_0 _values, suggesting that they might not be accurate mathematical models of the PCR process [37]. Furthermore, these models take into account the early PCR cycles that are swamped by fluorescence noise, leading to a large variation in the calculated *N*_0_. An additional explanation for the inadequate performance of the sigmoid, exponential, and LR *N*_0 _models is that they do not explicitly determine the efficiency value, and therefore cannot make use of average efficiencies obtained from several independent measurements. These observations thus support the conclusion that the determination of a precise efficiency value is paramount to the success of qPCR, and it provides a rational explanation for this phenomenon.

## Conclusion

Overall, three models stand out and may be used preferably depending on the experimental conditions and objectives: the Δ*Ct*, (*PavrgE*)^*Ct *^and (*SavrgE*)^*Ct *^models. Δ*Ct *will be preferred as an initial screening method when many different sequences have to be screened quickly and economically from few biological samples, but it will not provide precise estimates, either relative or absolute. (*PavrgE*)^*Ct *^and (*SavrgE*)^*Ct *^rely on an averaged efficiency value, either performed from all data resulting from one amplicon but irrespective of the biological sample or condition, or performed over each sample and amplicon, respectively. Thus, (*PavrgE*)^*Ct *^may be favoured when the same gene or sequence is to be amplified repeatedly from various biological treatments or specimens, or when following changes in the physiological or differentiation status of a cell population over time, to obtain comparative or relative estimates. In contrast, when absolute quantification of DNA and highest precision is needed, and/or when multiple sequences must be amplified from few biological samples or conditions, (*SavrgE*)^*Ct *^will be the method of choice, and the statistical analysis provided in this study will allow the estimation of the dataset size required to achieve a given accuracy.

## Methods

### Cell culture and cDNA preparation

Primary mouse fibroblast and NIH-3T3 mouse fibroblasts were cultured in DMEM supplemented with 10% serum. Cells were exposed to 100 pM TGF-β or to the ethanol carrier for 4 hours before RNA extraction. Total RNA was extracted from confluent 75 cm^2 ^culture dish (approx 2 million cells) using Trizol reagent (Invitrogen) according to the manufacturer's protocol and resuspended in 20 μl RNAse-free water. Reverse transcription was performed with the GeneAmp Gold RNA PCR Core kit (PE Applied Biosystem) using 5 μl (approx 2.5 μg) of RNA in a 25 μl final volume using oligo-dT as a primer. The resulting cDNA solution was diluted 10-fold in deionized water and the solution thus obtained was considered as the undiluted sample (1-fold dilution) for the qPCR measurements. This final dilution step was found to be necessary to prevent inhibitory effects on the PCR efficiency that likely result from contaminant carry-over (data not shown).

### Experimental set of qPCR data

To statistically qualify the quantitative PCR process and to evaluate the different models, we generated an experimental data set using 7 different amplicons. Expression of the genes listed in Additional File 1 is controlled by a regulatory cascade elicited by the treatment of fibroblastic cells with the Transforming Growth Factor-beta (TGF-β) growth factor. mRNAs from cells that were either untreated or induced by TGF-β were reverse transcribed to cDNA and evaluated by quantitative PCR. These primer pairs amplify portions of the Caveolin (Cav), Connective Tissue Growth Factor (CTGF), Elastin (Eln), Fibronectin (FN), Ribosomal protein L27, Perlecan (Perl) and Plasminogen Activator Inhibitor 1 (PAI-1) murine coding sequences. The primers were designed using the Primer Express 1.5a software (PE Applied Biosystem, Foster City, CA, USA) to generate amplicon size ranging between 51 and 149 base pairs (Additional File 1), and amplicons were located towards the 3' end of the coding sequence.

At least 4–8 distinct biological cDNA samples were used in conjunction with each of the 7 different target genes (amplicon). Each of these samples was serially diluted to obtain 10-fold, 50-fold, 100-fold and for some samples 1000-fold dilutions from the undiluted (1-fold) sample. Each of these dilutions was measured in 5 replicate PCR reactions (4 replicates for the 1000× dilutions), using each of the seven amplicons. This produced a data set of 704 reactions. The complete raw data set is given in the Additional File 2. Note that all sample were tested on the same PCR plate for a given amplicon. Thus we only addressed the intra-plate variability in this article and not the inter-plate variability.

### Quantitative PCR assays

SYBR green I technology was used for all quantitative PCR reactions, which were assembled using the Eurogentec kit RT-SN10-05 (Seraing, Belgium). Reactions were processed with 5.9 μl of cDNA samples in 25 μl final volume. One tip/well was used to distribute samples on the PCR plate in order to increase reproducibility of the data. Primers were all used at a final concentration of 100 nM and the specificity of the amplification product was verified for each reaction by examination of the corresponding dissociation curve. All PCR reactions were performed on an ABI Prism 7700 Sequence detector (PE Applied Biosystem, Foster City, CA, USA). For all reactions, cycling conditions were 95°C for 15 min (denaturation) and then 40 cycles of 95°C 15 sec – 62°C 1 min. Data acquisitions were performed with the SDS 1.9.1 software (PE Applied Biosystem, Foster City, CA, USA). Baseline limits were set as suggested by the manufacturer (i.e. at least two cycles before the rise of the earliest amplification). Threshold was set to lie in the middle of the exponential phase of the amplification plot, so that efficiency values truly reflect the reaction dynamic at the *Ct*. Unless otherwise noted in the text, all efficiency values were determined using the LinReg method [13]. Data resulting from reactions that did not reach the threshold within the first 40 cycles (*Ct *= 40) were discarded from the analysis.

### Equations

The full mathematical development of the following equations can be found in the Additional File 6.

The exponential behaviour of DNA increase in the exponential phase is described as follows:

*N*_*c *_= *N*_0 _· *E*^*c*^

where *N*_*c *_is the amount of PCR DNA product at cycle *c*; *N*_0 _the initial amount of target dsDNA and *E *the PCR reaction efficiency.

When comparing distinct samples, the relative DNA concentrations can be calculated as:

RAB=A0B0=EBCtBEACtA
 MathType@MTEF@5@5@+=feaafiart1ev1aaatCvAUfKttLearuWrP9MDH5MBPbIqV92AaeXatLxBI9gBaebbnrfifHhDYfgasaacH8akY=wiFfYdH8Gipec8Eeeu0xXdbba9frFj0=OqFfea0dXdd9vqai=hGuQ8kuc9pgc9s8qqaq=dirpe0xb9q8qiLsFr0=vr0=vr0dc8meaabaqaciaacaGaaeqabaqabeGadaaakeaacqWGsbGudaWgaaWcbaGaemyqaeKaemOqaieabeaakiabg2da9maalaaabaGaemyqae0aaSbaaSqaaiabicdaWaqabaaakeaacqWGcbGqdaWgaaWcbaGaeGimaadabeaaaaGccqGH9aqpdaWcaaqaaiabdweafnaaDaaaleaacqWGcbGqaeaacqWGdbWqcqWG0baDdaWgaaadbaGaemOqaieabeaaaaaakeaacqWGfbqrdaqhaaWcbaGaemyqaeeabaGaem4qamKaemiDaq3aaSbaaWqaaiabdgeabbqabaaaaaaaaaa@42C7@

where *R*_*AB *_represents the initial concentration ratio of sample A over B. Amplification efficiencies can be measured by taking the log of both side of Eq. 1, which gives a linear function of log *N*_*c *_= *f*(*c*):

log *N*_*c *_= log *N*_0 _+ *c *· log *E*

where the ordinate to the origin gives a direct estimate of *N*_0_, and the slope an estimate of the amplification efficiency. But in fact it must be noted that qPCR measures fluorescence that is proportional to the amount of DNA. Therefore Eq. 3 really measures *F*_0_, with *F*_0 _= *k*·*N*_0_. But this is not so important when measuring relative level of DNA since the ratio of initial fluorescence is equal to the initial ratio of target DNA.

When *c *= *C*_*t*_, Eq. 3 can be rearranged as:

Ct=−1log⁡E⋅log⁡N0+log⁡NCtlog⁡E
 MathType@MTEF@5@5@+=feaafiart1ev1aaatCvAUfKttLearuWrP9MDH5MBPbIqV92AaeXatLxBI9gBaebbnrfifHhDYfgasaacH8akY=wiFfYdH8Gipec8Eeeu0xXdbba9frFj0=OqFfea0dXdd9vqai=hGuQ8kuc9pgc9s8qqaq=dirpe0xb9q8qiLsFr0=vr0=vr0dc8meaabaqaciaacaGaaeqabaqabeGadaaakeaacqWGdbWqcqWG0baDcqGH9aqpcqGHsisldaWcaaqaaiabigdaXaqaaiGbcYgaSjabc+gaVjabcEgaNjabdweafbaacqGHflY1cyGGSbaBcqGGVbWBcqGGNbWzcqWGobGtdaWgaaWcbaGaeGimaadabeaakiabgUcaRmaalaaabaGagiiBaWMaei4Ba8Maei4zaCMaemOta40aaSbaaSqaaiabdoeadjabdsha0bqabaaakeaacyGGSbaBcqGGVbWBcqGGNbWzcqWGfbqraaaaaa@4E1D@

This expresses *Ct *as a linear function of (log *N*_0_), with a slope m=−1log⁡E
 MathType@MTEF@5@5@+=feaafiart1ev1aaatCvAUfKttLearuWrP9MDH5MBPbIqV92AaeXatLxBI9gBaebbnrfifHhDYfgasaacH8akY=wiFfYdH8Gipec8Eeeu0xXdbba9frFj0=OqFfea0dXdd9vqai=hGuQ8kuc9pgc9s8qqaq=dirpe0xb9q8qiLsFr0=vr0=vr0dc8meaabaqaciaacaGaaeqabaqabeGadaaakeaacqWGTbqBcqGH9aqpcqGHsisldaWcaaqaaiabigdaXaqaaiGbcYgaSjabc+gaVjabcEgaNjabdweafbaaaaa@3633@, allowing an estimation of a "mean" efficiency over all samples:

E=10−1m
 MathType@MTEF@5@5@+=feaafiart1ev1aaatCvAUfKttLearuWrP9MDH5MBPbIqV92AaeXatLxBI9gBaebbnrfifHhDYfgasaacH8akY=wiFfYdH8Gipec8Eeeu0xXdbba9frFj0=OqFfea0dXdd9vqai=hGuQ8kuc9pgc9s8qqaq=dirpe0xb9q8qiLsFr0=vr0=vr0dc8meaabaqaciaacaGaaeqabaqabeGadaaakeaacqWGfbqrcqGH9aqpcqaIXaqmcqaIWaamdaahaaWcbeqaaiabgkHiTmaaliaabaGaeGymaedabaGaemyBa0gaaaaaaaa@3422@

Alternatively, sigmoid fitting of amplification can be performed using Eq. 6

Nc=a1+exp⁡(x0−cb)
 MathType@MTEF@5@5@+=feaafiart1ev1aaatCvAUfKttLearuWrP9MDH5MBPbIqV92AaeXatLxBI9gBaebbnrfifHhDYfgasaacH8akY=wiFfYdH8Gipec8Eeeu0xXdbba9frFj0=OqFfea0dXdd9vqai=hGuQ8kuc9pgc9s8qqaq=dirpe0xb9q8qiLsFr0=vr0=vr0dc8meaabaqaciaacaGaaeqabaqabeGadaaakeaacqWGobGtdaWgaaWcbaGaem4yamgabeaakiabg2da9maalaaabaGaemyyaegabaGaeGymaeJaey4kaSIagiyzauMaeiiEaGNaeiiCaa3aaeWaaeaadaWcaaqaaiabdIha4naaBaaaleaacqaIWaamaeqaaOGaeyOeI0Iaem4yamgabaGaemOyaigaaaGaayjkaiaawMcaaaaaaaa@3F7C@

where *a*, *b *and *x*_0 _are fitting parameters and *c *is the cycle number. The original amount of target DNA is given by:

N0=a1+exp⁡(x0b)
 MathType@MTEF@5@5@+=feaafiart1ev1aaatCvAUfKttLearuWrP9MDH5MBPbIqV92AaeXatLxBI9gBaebbnrfifHhDYfgasaacH8akY=wiFfYdH8Gipec8Eeeu0xXdbba9frFj0=OqFfea0dXdd9vqai=hGuQ8kuc9pgc9s8qqaq=dirpe0xb9q8qiLsFr0=vr0=vr0dc8meaabaqaciaacaGaaeqabaqabeGadaaakeaacqWGobGtdaWgaaWcbaGaeGimaadabeaakiabg2da9maalaaabaGaemyyaegabaGaeGymaeJaey4kaSIagiyzauMaeiiEaGNaeiiCaa3aaeWaaeaadaWcaaqaaiabdIha4naaBaaaleaacqaIWaamaeqaaaGcbaGaemOyaigaaaGaayjkaiaawMcaaaaaaaa@3CDF@

Exponential fitting can also be performed but it requires to first trim the data, removing values that are in phase III. Then the remaining data can be fitted using:

*N*_*c *_= exp[*a *· (*c *- *x*_0_)]

where *a *and *x*_0 _are fitting parameters and *c *is the cycle number. The initial amount of target DNA is given by:

*N*_0 _= exp[-*a *· *x*_0_]

The propagation of error was determined using a Taylor expansion to the first order. For the first part of the normalized ratio (Eq. 2), this led to:

|ΔRAB|=RAB⋅(ΔA0A0)2+(ΔB0B0)2
 MathType@MTEF@5@5@+=feaafiart1ev1aaatCvAUfKttLearuWrP9MDH5MBPbIqV92AaeXatLxBI9gBaebbnrfifHhDYfgasaacH8akY=wiFfYdH8Gipec8Eeeu0xXdbba9frFj0=OqFfea0dXdd9vqai=hGuQ8kuc9pgc9s8qqaq=dirpe0xb9q8qiLsFr0=vr0=vr0dc8meaabaqaciaacaGaaeqabaqabeGadaaakeaadaabdaqaaiabfs5aejabdkfasnaaBaaaleaacqWGbbqqcqWGcbGqaeqaaaGccaGLhWUaayjcSdGaeyypa0JaemOuai1aaSbaaSqaaiabdgeabjabdkeacbqabaGccqGHflY1daGcaaqaamaabmaabaWaaSaaaeaacqqHuoarcqWGbbqqdaWgaaWcbaGaeGimaadabeaaaOqaaiabdgeabnaaBaaaleaacqaIWaamaeqaaaaaaOGaayjkaiaawMcaamaaCaaaleqabaGaeGOmaidaaOGaey4kaSYaaeWaaeaadaWcaaqaaiabfs5aejabdkeacnaaBaaaleaacqaIWaamaeqaaaGcbaGaemOqai0aaSbaaSqaaiabicdaWaqabaaaaaGccaGLOaGaayzkaaWaaWbaaSqabeaacqaIYaGmaaaabeaaaaa@4D72@

where Δ*R*_*AB *_is the standard deviation of the ration of amplicon A over amplicon B, and Δ*A*_0 _and Δ*B*_0 _the standard deviation of the initial amount of target DNA of amplicon A and B.

For the second part of the normalized ratio (Eq. 2), the propagation of errors is described by:

|ΔRAB|≅RAB⋅(CtAEA)2⋅(ΔEA)2+(ln⁡EA)2⋅(ΔCtA)2+(CtBEB)2⋅(ΔEB)2+(ln⁡EB)2⋅(ΔCtB)2
 MathType@MTEF@5@5@+=feaafiart1ev1aaatCvAUfKttLearuWrP9MDH5MBPbIqV92AaeXatLxBI9gBaebbnrfifHhDYfgasaacH8akY=wiFfYdH8Gipec8Eeeu0xXdbba9frFj0=OqFfea0dXdd9vqai=hGuQ8kuc9pgc9s8qqaq=dirpe0xb9q8qiLsFr0=vr0=vr0dc8meaabaqaciaacaGaaeqabaqabeGadaaakeaadaabdaqaaiabfs5aejabdkfasnaaBaaaleaacqWGbbqqcqWGcbGqaeqaaaGccaGLhWUaayjcSdGaeyyrIaKaemOuai1aaSbaaSqaaiabdgeabjabdkeacbqabaGccqGHflY1daGcaaqaamaabmaabaWaaSaaaeaacqWGdbWqcqWG0baDdaWgaaWcbaGaemyqaeeabeaaaOqaaiabdweafnaaBaaaleaacqWGbbqqaeqaaaaaaOGaayjkaiaawMcaamaaCaaaleqabaGaeGOmaidaaOGaeyyXIC9aaeWaaeaacqqHuoarcqWGfbqrdaWgaaWcbaGaemyqaeeabeaaaOGaayjkaiaawMcaamaaCaaaleqabaGaeGOmaidaaOGaey4kaSYaaeWaaeaacyGGSbaBcqGGUbGBcqWGfbqrdaWgaaWcbaGaemyqaeeabeaaaOGaayjkaiaawMcaamaaCaaaleqabaGaeGOmaidaaOGaeyyXIC9aaeWaaeaacqqHuoarcqWGdbWqcqWG0baDdaWgaaWcbaGaemyqaeeabeaaaOGaayjkaiaawMcaamaaCaaaleqabaGaeGOmaidaaOGaey4kaSYaaeWaaeaadaWcaaqaaiabdoeadjabdsha0naaBaaaleaacqWGcbGqaeqaaaGcbaGaemyrau0aaSbaaSqaaiabdkeacbqabaaaaaGccaGLOaGaayzkaaWaaWbaaSqabeaacqaIYaGmaaGccqGHflY1daqadaqaaiabfs5aejabdweafnaaBaaaleaacqWGcbGqaeqaaaGccaGLOaGaayzkaaWaaWbaaSqabeaacqaIYaGmaaGccqGHRaWkdaqadaqaaiGbcYgaSjabc6gaUjabdweafnaaBaaaleaacqWGcbGqaeqaaaGccaGLOaGaayzkaaWaaWbaaSqabeaacqaIYaGmaaGccqGHflY1daqadaqaaiabfs5aejabdoeadjabdsha0naaBaaaleaacqWGcbGqaeqaaaGccaGLOaGaayzkaaWaaWbaaSqabeaacqaIYaGmaaaabeaaaaa@8555@

Standard deviation on the efficiencies calculated with the Serial dilution were evaluated from the standard deviation of the slope of the regression using a Taylor expansion to the first order for error propagation:

|ΔE|=(E⋅ln⁡10⋅Δm)2
 MathType@MTEF@5@5@+=feaafiart1ev1aaatCvAUfKttLearuWrP9MDH5MBPbIqV92AaeXatLxBI9gBaebbnrfifHhDYfgasaacH8akY=wiFfYdH8Gipec8Eeeu0xXdbba9frFj0=OqFfea0dXdd9vqai=hGuQ8kuc9pgc9s8qqaq=dirpe0xb9q8qiLsFr0=vr0=vr0dc8meaabaqaciaacaGaaeqabaqabeGadaaakeaadaabdaqaaiabfs5aejabdweafbGaay5bSlaawIa7aiabg2da9maakaaabaGaeiikaGIaemyrauKaeyyXICTagiiBaWMaeiOBa4MaeGymaeJaeGimaaJaeyyXICTaeuiLdqKaemyBa0MaeiykaKYaaWbaaSqabeaacqaIYaGmaaaakeqaaaaa@434C@

where Δ*E *is the standard deviation on the efficiency and Δ*m *is the standard deviation of the slope of the regression.

Standard deviation on the efficiencies measured with LinReg were obtained by averaging all efficiencies obtained from the same data set used for the Serial dilution.

When comparing the expression of a gene in different experimental conditions, the useful figure is the normalized induction ratio:

I1−2=RAB(1)RAB(2)
 MathType@MTEF@5@5@+=feaafiart1ev1aaatCvAUfKttLearuWrP9MDH5MBPbIqV92AaeXatLxBI9gBaebbnrfifHhDYfgasaacH8akY=wiFfYdH8Gipec8Eeeu0xXdbba9frFj0=OqFfea0dXdd9vqai=hGuQ8kuc9pgc9s8qqaq=dirpe0xb9q8qiLsFr0=vr0=vr0dc8meaabaqaciaacaGaaeqabaqabeGadaaakeaacqWGjbqsdaWgaaWcbaGaeGymaeJaeyOeI0IaeGOmaidabeaakiabg2da9maalaaabaGaemOuai1aaSbaaSqaaiabdgeabjabdkeacjabcIcaOiabigdaXiabcMcaPaqabaaakeaacqWGsbGudaWgaaWcbaGaemyqaeKaemOqaiKaeiikaGIaeGOmaiJaeiykaKcabeaaaaaaaa@3E14@

where *I*_1–2 _is the ratio of the expression of the gene of interest (induction) between condition 1 and condition 2 and *R*_*AB(i) *_is the normalized expression of the gene interest (Eq. 2) in condition *i *(1 or 2). Note that Eq. 13 is valid only if the gene used for the normalization (internal standard) has an expression that is invariant with condition 1 and 2 [38]. Error on induction values is given by

ΔI1−2=I1−2⋅(ΔRAB(1)RAB(1))2+(ΔRAB(2)RAB(2))2
 MathType@MTEF@5@5@+=feaafiart1ev1aaatCvAUfKttLearuWrP9MDH5MBPbIqV92AaeXatLxBI9gBaebbnrfifHhDYfgasaacH8akY=wiFfYdH8Gipec8Eeeu0xXdbba9frFj0=OqFfea0dXdd9vqai=hGuQ8kuc9pgc9s8qqaq=dirpe0xb9q8qiLsFr0=vr0=vr0dc8meaabaqaciaacaGaaeqabaqabeGadaaakeaacqqHuoarcqWGjbqsdaWgaaWcbaGaeGymaeJaeyOeI0IaeGOmaidabeaakiabg2da9iabdMeajnaaBaaaleaacqaIXaqmcqGHsislcqaIYaGmaeqaaOGaeyyXIC9aaOaaaeaadaqadaqaamaalaaabaGaeuiLdqKaemOuai1aaSbaaSqaaiabdgeabjabdkeacjabcIcaOiabigdaXiabcMcaPaqabaaakeaacqWGsbGudaWgaaWcbaGaemyqaeKaemOqaiKaeiikaGIaeGymaeJaeiykaKcabeaaaaaakiaawIcacaGLPaaadaahaaWcbeqaaiabikdaYaaakiabgUcaRmaabmaabaWaaSaaaeaacqqHuoarcqWGsbGudaWgaaWcbaGaemyqaeKaemOqaiKaeiikaGIaeGOmaiJaeiykaKcabeaaaOqaaiabdkfasnaaBaaaleaacqWGbbqqcqWGcbGqcqGGOaakcqaIYaGmcqGGPaqkaeqaaaaaaOGaayjkaiaawMcaamaaCaaaleqabaGaeGOmaidaaaqabaaaaa@5B52@

See Additional File 6 for the full mathematical development of Eq. 1 to Eq. 14.

Finally, the number of replicate needed to reach statistical significance can be calculated as follows:

n=(CV⋅Imeas)2(z1−α2)2(Range⋅Imeas)2=CV2(z1−α2)2(Range)2
 MathType@MTEF@5@5@+=feaafiart1ev1aaatCvAUfKttLearuWrP9MDH5MBPbIqV92AaeXatLxBI9gBaebbnrfifHhDYfgasaacH8akY=wiFfYdH8Gipec8Eeeu0xXdbba9frFj0=OqFfea0dXdd9vqai=hGuQ8kuc9pgc9s8qqaq=dirpe0xb9q8qiLsFr0=vr0=vr0dc8meaabaqaciaacaGaaeqabaqabeGadaaakeaacqWGUbGBcqGH9aqpdaWcaaqaamaabmaabaGaem4qamKaemOvayLaeyyXICTaemysaK0aaSbaaSqaaiabd2gaTjabdwgaLjabdggaHjabdohaZbqabaaakiaawIcacaGLPaaadaahaaWcbeqaaiabikdaYaaakmaabmaabaGaemOEaO3aaSbaaSqaaiabigdaXiabgkHiTmaaliaabaGaeqySdegabaGaeGOmaidaaaqabaaakiaawIcacaGLPaaadaahaaWcbeqaaiabikdaYaaaaOqaamaabmaabaGaemOuaiLaemyyaeMaemOBa4Maem4zaCMaemyzauMaeyyXICTaemysaK0aaSbaaSqaaiabd2gaTjabdwgaLjabdggaHjabdohaZbqabaaakiaawIcacaGLPaaadaahaaWcbeqaaiabikdaYaaaaaGccqGH9aqpdaWcaaqaaiabdoeadjabdAfawnaaCaaaleqabaGaeGOmaidaaOWaaeWaaeaacqWG6bGEdaWgaaWcbaGaeGymaeJaeyOeI0YaaSGaaeaacqaHXoqyaeaacqaIYaGmaaaabeaaaOGaayjkaiaawMcaamaaCaaaleqabaGaeGOmaidaaaGcbaWaaeWaaeaacqWGsbGucqWGHbqycqWGUbGBcqWGNbWzcqWGLbqzaiaawIcacaGLPaaadaahaaWcbeqaaiabikdaYaaaaaaaaa@6EE8@

where n is the number of independent replicates, z1−α2
 MathType@MTEF@5@5@+=feaafiart1ev1aaatCvAUfKttLearuWrP9MDH5MBPbIqV92AaeXatLxBI9gBaebbnrfifHhDYfgasaacH8akY=wiFfYdH8Gipec8Eeeu0xXdbba9frFj0=OqFfea0dXdd9vqai=hGuQ8kuc9pgc9s8qqaq=dirpe0xb9q8qiLsFr0=vr0=vr0dc8meaabaqaciaacaGaaeqabaqabeGadaaakeaacqWG6bGEdaWgaaWcbaGaeGymaeJaeyOeI0YaaSGaaeaacqaHXoqyaeaacqaIYaGmaaaabeaaaaa@32D5@ the normalized reduced value related to significance level α of the statistical test (here α was set to 0.05, which corresponds to Z = 1.96) and *CV *the coefficient of variation related to the measured inductions. *Range *is defined as the largest acceptable error on the measure, and it is set arbitrarily by the experimenter. See Additional File 7 for the full development of Eq. 15.

## Authors' contributions

YK carried out the qPCR experiment, performed all of the statistical tests and wrote the article. AMN carried out some qPCR experiment. SP programmed the macro for the non-linear fitting of the amplification plots. CM provided the mathematical validation of the LinReg method. NM provided oversight of the work and helped finalize the article.

## Supplementary Material

Additional file 1 **Additional tables**. Additional Table 1: Primer sequences and qPCR dataset description. Additional Table 2: Mean efficiency of each primer set. Additional Table 3: Induction ratio of extracellular matrix gene by TGF-β as assessed from 10 replicate assays. Additional Table 4: Induction ratio of extracellular matrix gene by TGF-β as assessed from 3 replicate assays Click here for file

Additional file 2 **Complete set of data and macro**. Excel file containing all raw qPCR data and the macro used into the present article. Click here for file

Additional file 3 **Additional Figures**. Additional Figure 1: Reproducibility of *Ct *measurements. Additional Figure 2: Precision and Robustness of the different models. Click here for file

Additional file 4 **Mathematical Justification of LinReg**. Justification of the LinReg method to estimate PCR efficiency, when PCR is considered as a branching process. Click here for file

Additional file 5 **ΔCt systematic bias**. When not fulfilled, the Δ*Ct *assumption of equal efficiency induces a bias in induction estimates. Equations are developed to estimate the bias as a function of the real efficiency. Click here for file

Additional file 6 **Equation development**. Detailed development of all equations 1–14 of the Methods section. Click here for file

Additional file 7 **Statistical significance and required sample size**. Presentation of all of the equations leading to the development of eq.15 of the Methods section. Click here for file
