# Supplementary material for: Statistical significance of quantitative PCR
Source: BMC Bioinformatics. 2007 Apr 20;8:131. doi: 10.1186/1471-2105-8-131 (PMC1868764; doi:10.1186/1471-2105-8-131)
Supplement: Additional file 1 — Additional tables. Additional Table 1: Primer sequences and qPCR dataset description. Additional Table 2: Mean efficiency of each primer set. Additional Table 3: Induction ratio of extracellular matrix gene by TGF-β as assessed from 10 replicate assays. Additional Table 4: Induction ratio of extracellular matrix gene by TGF-β as assessed from 3 replicate assays [file 1471-2105-8-131-S1.pdf]

# Statistical significance of quantitative PCR: Additional File 1

Yann Karlen[1], Alan McNair[1], Sébastien Perseguer[2], Christian Mazza[3] and Nicolas Mermod[1]*

## Additional Tables

**Additional Table1: Primer sequences and qPCR dataset**

| Gene | Forward primer | Reverse primer | Amplicon size | Samples | Dilutions |
|---|---|---|---|---|---|
| Cav | TGATGAGTGAACCTCCAGGGA | GCAGTCTCGGTTTAGCAGCC | 57 | 4 | 4 |
| CTGF | CATTAAGAAGGGCAAAAAGTGCA | ACAGGCTTGGCGATTTTAGGT | 51 | 4 | 5 |
| Eln | TCTTTGTGTTTCGCTGTGATAGATC | CAAACATCATCCCCAAATATCCA | 72 | 4 | 4 |
| FN | GCAAGGAAAGTCACCCAGACA | TGCAGGTCAGATGGCAAAAG | 51 | 4 | 5 |
| L27 | GCCAAGCGATCCAAGATCAA | GCTGGGTCCCTGAACACATC | 120 | 8 | 5 |
| Perl | GGAGGCCCGTCTTGTCTCA | TGTTGACCGCCACATTAGGAC | 149 | 4 | 4 |
| PAI-1 | CCCAATAGCGAGCCTTCTCC | TGTGGGTCGAAAAAAGCTGTG | 100 | 4 | 4 |

Target genes, primer sequences and amplicon size (in base pair, primers included). The number of independent biological samples used for each primer set and number of dilutions used in the serial dilution assay is indicated. Each dilution was measured in 5 replicates PCR. The size of the messenger RNA is indicated only for the genes analyzed in the Northern blot assay.

**Additional Table 2: Average efficiencies measured for each set of primers**

| Primer set | Efficiency | SEM | n |
|---|---|---|---|
| Cav | 1.676 | 0.0088 | 80 |
| CTGF | 1.843 | 0.0055 | 97 |
| Eln | 1.753 | 0.0062 | 80 |
| FN | 1.827 | 0.0064 | 116 |
| L27 | 1.911 | 0.0040 | 211 |
| Perl | 1.753 | 0.0111 | 76 |
| PAI-1 | 1.896 | 0.0080 | 100 |

Efficiencies were determined using the LinReg method for each target cDNA using the dataset described in Add. Table 1, and they are expressed as the average, standard deviation of the mean (SEM), and number of values used for each determination

Additional Table 3: TGF-β induction of extracellular matrix gene expression in NIH-3T3 fibroblasts as assessed from 10 replicate assays

| Gene | PAI-1 | | | FN | | | CTGF | | |
|---|---|---|---|---|---|---|---|---|---|
| | $(\text{Savrg E})^{Ct}$ | $(\text{Pavrg E})^{Ct}$ | $\Delta$Ct | $(\text{Savrg E})^{Ct}$ | $(\text{Pavrg E})^{Ct}$ | $\Delta$Ct | $(\text{Savrg E})^{Ct}$ | $(\text{Pavrg E})^{Ct}$ | $\Delta$Ct |
| Induction | 8.01 | 14.75 | 14.11 | 1.60 | 1.08 | 1.08 | 23.15 | 43.53 | 40.96 |
| SD | 1.12 | 2.09 | 1.98 | 0.21 | 0.14 | 0.14 | 3.21 | 6.46 | 6.01 |
| CV | 14.04 | 14.17 | 14.04 | 12.93 | 13.37 | 13.25 | 13.84 | 14.83 | 14.68 |
| p-value | <0.0001 | <0.0001 | <0.0001 | 0.03 | 0.384 | 0.389 | <0.0001 | <0.0001 | <0.0001 |

The expression of the PAI1, FN and CTGF genes was measured and normalized to the L27 mRNA using the $(SavrgE)^{Ct}$, $(PavrgE)^{Ct}$ and the $\Delta Ct$ models. Average induction ratios (e.g. induced over non-induced cDNA ratios) were determined over the entire set of 10 replicate assays and they are shown alongside with the standard deviation and coefficient of variation (CV) values. A t-test was performed on the normalized gene expression to test whether expression levels are statistically different between the induced and non-induced state. A p-value below 0.05 indicates that gene expression levels are statistically different.

Additional Table 4: TGF-β induction of extracellular matrix gene expression in NIH-3T3 fibroblasts as assessed from 3 replicate assays

| Gene | PAI-1 | | | FN | | | CTGF | | |
|---|---|---|---|---|---|---|---|---|---|
| | $(Savrg\ E)^{Ct}$ | $(Pavrg\ E)^{Ct}$ | ΔCt | $(Savrg\ E)^{Ct}$ | $(Pavrg\ E)^{Ct}$ | ΔCt | $(Savrg\ E)^{Ct}$ | $(Pavrg\ E)^{Ct}$ | ΔCt |
| Induction | 23.64 | 13.74 | 12.94 | 2.38 | 1.07 | 1.09 | 44.01 | 34.23 | 44.12 |
| SD | 3.84 | 2.22 | 2.05 | 0.26 | 0.11 | 0.12 | 4.95 | 3.86 | 5.29 |
| CV | 16.25 | 16.17 | 15.86 | 10.75 | 10.59 | 11.25 | 11.25 | 11.29 | 11.99 |
| p-value | <0.0001 | <0.0001 | <0.0001 | <0.0001 | 0.263 | 0.236 | <0.0001 | <0.0001 | <0.0001 |

Expression of 3 genes was measured and normalized with L27 using the $(SavrgE)^{Ct}$, $(PavrgE)^{Ct}$ and the $\Delta Ct$ models, and results are represented as described in the footnote to Additional Table 3, except that only the first three measurements were taken into account.
