# Supplementary material for: Statistical significance of quantitative PCR
Source: BMC Bioinformatics. 2007 Apr 20;8:131. doi: 10.1186/1471-2105-8-131 (PMC1868764; doi:10.1186/1471-2105-8-131)
Supplement: Additional file 2 — Complete set of data and macro. Excel file containing all raw qPCR data and the macro used into the present article. [file 1471-2105-8-131-S2.ZIP › SigmoidFit.rtf]

Option Explicit
Option Base 1

' _____________________________________________________________________________
'
' SIGMOID FIT
'
' Sébastien Perseguers, june 2005
'
' Description : Interpolate a set of values (X,Y) according to the function
'
'               Y = A / (1 + exp((X0 - X) / B))
' _____________________________________________________________________________


' *****************************************************************************
' CONSTANTS AND VARIABLES DECLARATION
' *****************************************************************************

Private Const PRECISION = 0.00001
Private Const R2_MIN = 0.5          ' the interpolation failed if R2 < R2_MIN

Private DataX() As Single
Private DataY() As Single
Private DataMax As Single
Private Dist0 As Single             ' needed for R_Squared_Value
Private Param(1 To 3) As Single     ' (A, B, X0)
Private ParamIni(1 To 3) As Single
Private Grad(1 To 3) As Single      ' (GradA, GradB, GradX0)

' *****************************************************************************
' PROCEDURES
' *****************************************************************************

' -----------------------------------------------------------------------------
' Name : SigmoidFit
' Desc : For each set of values in the worksheet, search the best interpolation
' -----------------------------------------------------------------------------
Sub SigmoidFit()
'
' SigmoidFit Macro
' Macro recorded 10.06.2004 by Sébastien Perseguers
'
Dim i As Integer, j As Integer
Dim ColumnMax As Integer
Dim R2 As Single
Dim strFailed As String

    ' number of data for each interpolation
    DataMax = 0
    While Cells(DataMax + 2, 1).value <> ""
        DataMax = DataMax + 1
    Wend
    
    ' number of interpolations
    ColumnMax = 0
    While Cells(1, ColumnMax + 2).value <> ""
        ColumnMax = ColumnMax + 1
    Wend

    ' move all cells : place is needed to show the results
    range(Cells(1, 1), Cells(DataMax + 1, ColumnMax + 1)).Cut range("A6")
    range("A1").value = "a"         ' A
    range("A2").value = "b"         ' B
    range("A3").value = "x0"        ' C
    range("A4").value = "R^2"
    range("A5").value = "F0"

    ' load DataX and resize the array
    ReDim DataX(DataMax)
    ReDim DataY(DataMax)
    For i = 1 To DataMax
        DataX(i) = Cells(i + 6, 1).value
    Next
        
    ' ask for the initial values
    ParamIni(1) = InputBox("Initial value for 'a' :", "Sigmoid Fit", 1)
    ParamIni(2) = InputBox("Initial value for 'b' :", "Sigmoid Fit", 1)
    ParamIni(3) = InputBox("Initial value for 'x0' :", "Sigmoid Fit", Int(DataMax / 2))
    Param(1) = ParamIni(1)
    Param(2) = ParamIni(2)
    Param(3) = ParamIni(3)
    
    ' interpolate the data for each column
    R2 = 0
    strFailed = ""
    range("A1").Activate
    For i = 1 To ColumnMax
        ' load DataY
        Dist0 = 0
        For j = 1 To DataMax
            DataY(j) = Cells(j + 6, i + 1).value
            Dist0 = Dist0 + DataY(j) ^ 2
        Next
        Dist0 = Sqr(Dist0)
        ' do the interpolation and show the result
        R2 = FitData
        If R2 < R2_MIN Then             ' interpolation failed
            strFailed = strFailed & Cells(6, i + 1) & " "
            Param(1) = ParamIni(1)
            Param(2) = ParamIni(2)
            Param(3) = ParamIni(3)
        Else
            Cells(1, i + 1).value = Param(1)
            Cells(2, i + 1).value = Param(2)
            Cells(3, i + 1).value = Param(3)
            Cells(4, i + 1).value = FitData
            Cells(5, i + 1).value = Sigmoid(0)
        End If
        ' do not panic, the program does not sleep !
        DoEvents
        range("A1").value = CStr(Int(i / ColumnMax * 100)) & " %"
    Next
    range("A1").value = "a"
    
    If strFailed = "" Then
        MsgBox "Done", vbInformation, "Sigmoïd Fit"
    Else
        MsgBox "The following interpolations failed : " _
               & vbNewLine & strFailed, vbInformation, "Sigmoïd Fit"
    End If

End Sub

' -----------------------------------------------------------------------------
' Name : Sigmoid
' -----------------------------------------------------------------------------
Private Function Sigmoid(ByVal X As Single) As Single
    Sigmoid = Param(1) / (1 + Exp((Param(3) - X) / Param(2)))
End Function

' -----------------------------------------------------------------------------
' Name : Distance
' Desc : Return the sum of squared distance between DataY and interpolated Y
' -----------------------------------------------------------------------------
Private Function Distance() As Single
Dim Dist As Single
Dim i As Integer

    Dist = 0
    For i = 1 To DataMax
        Dist = Dist + (DataY(i) - Sigmoid(DataX(i))) ^ 2
    Next
    Distance = Sqr(Dist)
    
End Function

' -----------------------------------------------------------------------------
' Name : GetGradients
' Rem  : Return a normalized gradient : ||Grad|| = 1
' -----------------------------------------------------------------------------
Private Sub GetGradients()
Dim i As Integer
Dim Norm As Single
Dim T1 As Double, T2 As Double, T3 As Double

    Grad(1) = 0
    Grad(2) = 0
    Grad(3) = 0

    For i = 1 To DataMax
        T1 = Exp((Param(3) - DataX(i)) / Param(2))
        T2 = 2 * (DataY(i) - Param(1) / (1 + T1))
        T3 = T2 * Param(1) * T1 / (1 + T1) ^ 2
        Grad(1) = Grad(1) - T2 / (1 + T1)
        Grad(2) = Grad(2) - T3 * (Param(3) - DataX(i)) / Param(2) ^ 2
        Grad(3) = Grad(3) + T3 / Param(2)
    Next

    Norm = Sqr(Grad(1) ^ 2 + Grad(2) ^ 2 + Grad(3) ^ 2)
    Grad(1) = Grad(1) / Norm
    Grad(2) = Grad(2) / Norm
    Grad(3) = Grad(3) / Norm

End Sub

' -----------------------------------------------------------------------------
' Name : FitData
' Desc : Interpolate the sigmoid and return the R_Squared_Value
' Rem  : Take the previous values (A, B, X0) for the initial parameters,
'        unless the previous interpolation failed
' -----------------------------------------------------------------------------
Public Function FitData() As Single
Dim i As Integer
Dim OldDist As Single, Dist As Single
Dim OldGrad(1 To 3) As Single
Dim DParam(1 To 3) As Single
Dim DP As Single
Dim bEnd As Boolean
    
    ' initial values
    For i = 1 To 3
        DParam(i) = Param(i) / 20
        Grad(i) = 0
    Next
    
    Dist = 0
    Do
        ' new values
        For i = 1 To 3
            OldGrad(i) = Grad(i)
        Next
        GetGradients
        OldDist = Dist
        Dist = Distance

        ' no amelioration
        If Abs(Dist - OldDist) / Dist < PRECISION Then
            DP = 0.5
        Else
            DP = 1
        End If

        ' new variations and new parameters value
        bEnd = True
        For i = 1 To 3
            DParam(i) = DP * DParam(i) * (1 + 0.5 * Sgn(OldGrad(i) * Grad(i)))
            Param(i) = Param(i) - DParam(i) * Grad(i)
            ' end condition
            If DParam(i) > Abs(Param(i)) * PRECISION Then bEnd = False
        Next
        
    Loop Until bEnd
    
    ' R_Squared_Value
    FitData = (1 - Dist / Dist0) ^ 2
    
End Function
