# Supplementary material for: Statistical significance of quantitative PCR
Source: BMC Bioinformatics. 2007 Apr 20;8:131. doi: 10.1186/1471-2105-8-131 (PMC1868764; doi:10.1186/1471-2105-8-131)
Supplement: Additional file 2 — Complete set of data and macro. Excel file containing all raw qPCR data and the macro used into the present article. [file 1471-2105-8-131-S2.ZIP › ExpoFit.rtf]

Option Explicit
Option Base 1

' _____________________________________________________________________________
'
' EXPO FIT
'
' Sébastien Perseguers, june 2005
'
' Description : Interpolate a set of values (X,Y) according to the function
'
'               Y = A * exp(BX)
' _____________________________________________________________________________

' *****************************************************************************
' CONSTANTS AND VARIABLES DECLARATION
' *****************************************************************************

Private Const PRECISION = 0.00001

Private Const BMIN = 0.01
Private Const BMAX = 1

Private DataX() As Double
Private DataY() As Double
Private DataMax As Integer
Private N As Integer
Private Dist0 As Double             ' needed for R_Squared_Value
Private a As Double, b As Double

' *****************************************************************************
' PROCEDURES
' *****************************************************************************

' -----------------------------------------------------------------------------
' Name : ExpoFit (Macro)
' Desc : For each set of values in the worksheet, search the best interpolation
' -----------------------------------------------------------------------------
Sub ExpoFit()
Dim Threshold As Double                     ' threshold
Dim i As Integer, j As Integer
Dim ColumnMax As Integer
Dim RowStart As Integer
Dim R2 As Double

    ' number of data x
    DataMax = 0
    While Cells(DataMax + 2, 1).value <> ""
        DataMax = DataMax + 1
    Wend
    
    ' number of interpolations
    ColumnMax = 0
    While Cells(1, ColumnMax + 2).value <> ""
        ColumnMax = ColumnMax + 1
    Wend

    ' move all cells : place is needed to show the results
    range(Cells(1, 1), Cells(DataMax + 1, ColumnMax + 1)).Cut range("A6")
    range("A1").value = "A"
    range("A2").value = "X0"
    range("A3").value = "F0"
    range("A4").value = "R^2"
    range("A5").value = "n"
    RowStart = 6
    
    ' load DataX and resize the array
    ReDim DataX(DataMax)
    ReDim DataY(DataMax)
    For i = 1 To DataMax
        DataX(i) = Cells(i + RowStart, 1).value
    Next
            
    ' ask for the threshold
    Threshold = InputBox("Entrez la valeur de seuil :")
    
    ' interpolate the data for each column
    range("A1").Activate
    b = 0.5
    For i = 1 To ColumnMax
        ' load DataY
        Dist0 = 0
        N = DataMax
        For j = 1 To DataMax
            If Cells(j + RowStart, i + 1).value > Threshold Then
                N = j - 1
                Exit For
            End If
            DataY(j) = Cells(j + RowStart, i + 1).value
            Dist0 = Dist0 + DataY(j) ^ 2
        Next
        Dist0 = Sqr(Dist0)
        ' do the interpolation and show the result
        R2 = FitData
        If R2 > 0 And a > 0 Then
            Cells(1, i + 1).value = b
            Cells(2, i + 1).value = -Log(a) / b
            Cells(3, i + 1).value = a
            Cells(4, i + 1).value = R2
            Cells(5, i + 1).value = N
        Else
            ' interpolation failed
            Cells(1, i + 1).value = "Failed"
        End If
NextColumn:
    Next
    
End Sub

' -----------------------------------------------------------------------------
' Name : GetA
' Desc : A is a function of B
' -----------------------------------------------------------------------------
Private Function GetA(ByVal valB As Double) As Double
Dim i As Integer
Dim Sum1 As Double: Sum1 = 0
Dim Sum2 As Double: Sum2 = 0

    For i = 1 To N
        Sum1 = Sum1 + Exp(valB * DataX(i)) * DataY(i)
        Sum2 = Sum2 + Exp(valB * DataX(i) * 2)
    Next
    
    GetA = Sum1 / Sum2
    
End Function

' -----------------------------------------------------------------------------
' Name : GetDA
' Desc : Return the derivative of A
' -----------------------------------------------------------------------------
Private Function GetDA(ByVal valB As Double) As Double
Dim i As Integer
Dim Sum(1 To 4) As Double
Dim X As Double, Y As Double
Dim e1 As Double, e2 As Double

    For i = 1 To 4
        Sum(i) = 0
    Next
    For i = 1 To N
        X = DataX(i)
        Y = DataY(i)
        e1 = Exp(valB * X)
        e2 = Exp(valB * X * 2)
        Sum(1) = Sum(1) + X * Y * e1
        Sum(2) = Sum(2) + e2
        Sum(3) = Sum(3) + Y * e1
        Sum(4) = Sum(4) + 2 * X * e2
    Next
    GetDA = (Sum(1) * Sum(2) - Sum(3) * Sum(4)) / Sum(2) ^ 2
    
End Function

' -----------------------------------------------------------------------------
' Name : Root
' Desc : It is the function whose root is searched
' -----------------------------------------------------------------------------
Private Function Root(ByVal valB As Double) As Double
Dim i As Integer
Dim Sum As Double
Dim X As Double, Y As Double
Dim valA As Double

    valA = GetA(valB)
    
    Sum = 0
    For i = 1 To N
        X = DataX(i)
        Y = DataY(i)
        Sum = Sum + X * valA * (valA * Exp(2 * valB * X) - Y * Exp(valB * X))
    Next
    Root = Sum
    
End Function

' -----------------------------------------------------------------------------
' Name : DRoot
' Desc : d Root / d B
' -----------------------------------------------------------------------------
Private Function DRoot(ByVal valB As Double) As Double
Dim i As Integer
Dim Sum As Double
Dim X As Double, Y As Double
Dim valA As Double, valDA As Double

    valA = GetA(valB)
    valDA = GetDA(valB)
    
    Sum = 0
    For i = 1 To N
        X = DataX(i)
        Y = DataY(i)
        Sum = Sum + X * (valDA + valA * X) * _
                   (2 * valA * Exp(2 * valB * X) - Y * Exp(valB * X))
    Next
    DRoot = Sum
    
End Function

' -----------------------------------------------------------------------------
' Name : FitData
' Desc : Use Newton's method to find B
' -----------------------------------------------------------------------------
Private Function FitData() As Double
Dim OldB As Double
Dim i As Integer
Dim dB As Double
Dim Dist As Double

    ' first approximation for B
    dB = (BMAX - BMIN) / 20
    For i = 0 To 20
        b = BMIN + i * dB
        If Root(b) > 0 Then Exit For
    Next
    
    b = b - dB / 2
    a = GetA(b)

    Do
        OldB = b
        b = OldB - Root(OldB) / DRoot(OldB)
        ' the interpolation failed
        If b > BMAX Or b < BMIN Then
            FitData = -1
            Exit Function
        End If
    Loop Until Abs((b - OldB) / b) < PRECISION
    a = GetA(b)
    
    ' R_Squared_Value
    Dist = 0
    For i = 1 To N
        Dist = Dist + (DataY(i) - a * Exp(b * DataX(i))) ^ 2
    Next
    Dist = Sqr(Dist)
    FitData = (1 - Dist / Dist0) ^ 2
    
End Function
