# Supplementary material for: Statistical significance of quantitative PCR
Source: BMC Bioinformatics. 2007 Apr 20;8:131. doi: 10.1186/1471-2105-8-131 (PMC1868764; doi:10.1186/1471-2105-8-131)
Supplement: Additional file 3 — Additional Figures. Additional Figure 1: Reproducibility of Ct measurements. Additional Figure 2: Precision and Robustness of the different models. [file 1471-2105-8-131-S3.pdf]

Statistical significance of quantitative PCR: Additional File 3

Yann Karlen[1], Alan McNair[1], Sébastien Perseguer[2], Christian Mazza[3] and Nicolas Mermod[1]*

Additional figures 1 and 2

Additional Figure 1: Reproducibility of Ct measurements.

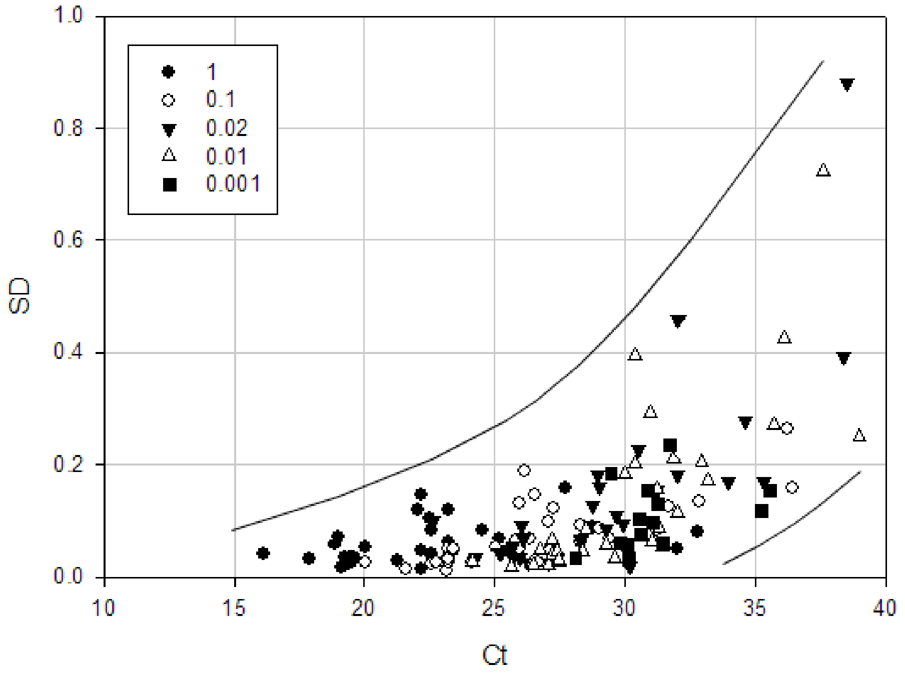

Additional Figure 1: Reproducibility of Ct measurements.

144 distinct PCR samples were generated using various cDNA isolates, primers and dilutions, as described in the Materials and Methods section. Four or five replicate PCR reactions were performed from each sample. The average *Ct* calculated for each PCR sample is represented as a function of its associated standard deviation (SD). Data symbol represent dilutions of the original samples, as described in the legend to Figure 1.

Interestingly, Larionov and colleagues have shown that for normally distributed *Ct* with associated CV value below 1%, the data processing of the *Ct* leave the resulting figures normally distributed, which allows the use of classical statistics [1]. Thus, all replicates with CV above 1% were excluded from the dataset, which corresponds to some of the reactions with *Ct* above 30 in this study.

Additional Figure 2: Precision and Robustness of the different models.

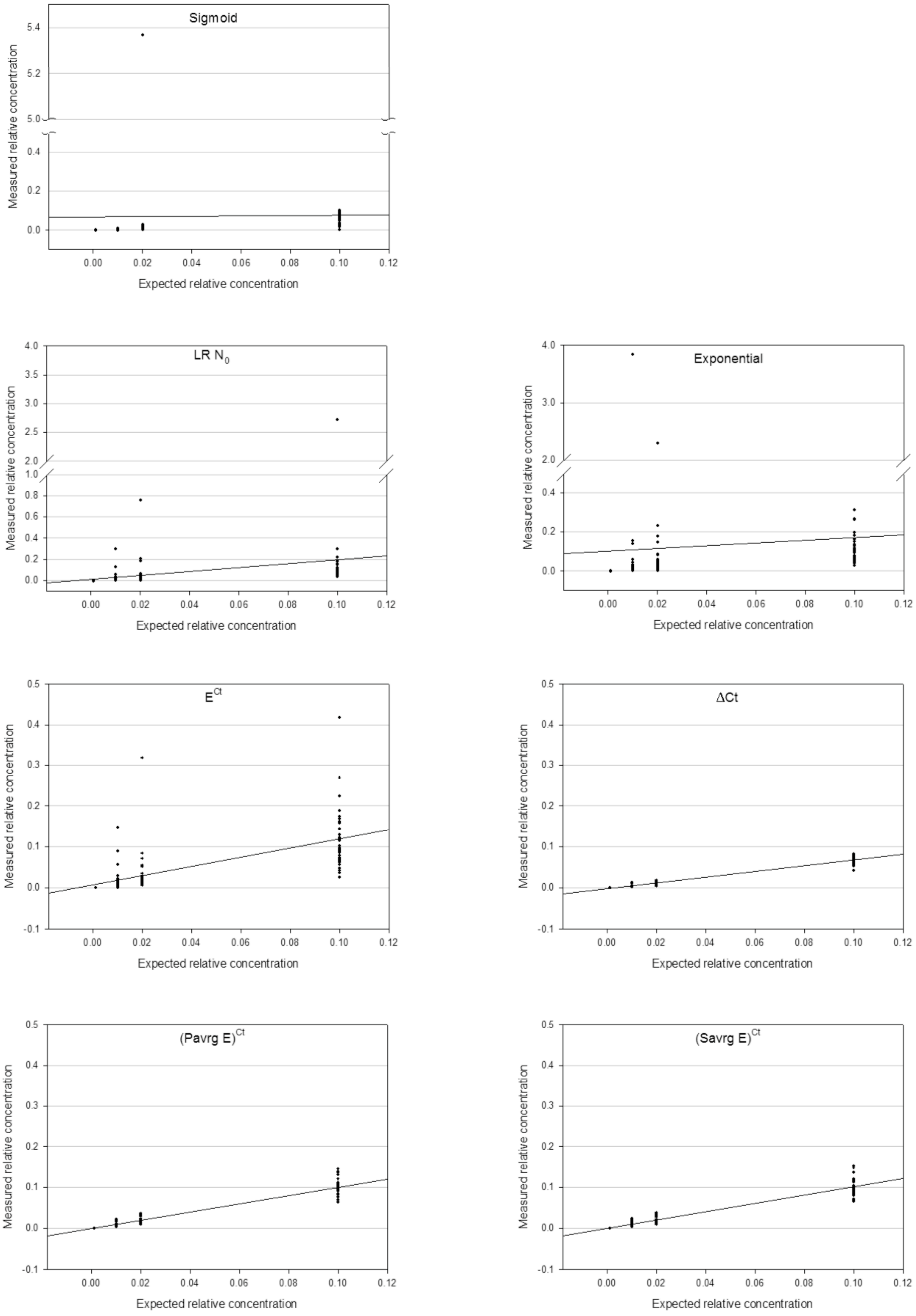

Additional Figure 2: Precision and Robustness of the different models.

Normalized measured DNA concentration ratio (primer, sample) are represented for each model relative to the concentration expected from the sample dilution ratio. A linear regression through all points was performed. Slope and the r^2 values of the linear regression close to 1 indicate high precision and robustness, respectively (Table 4).

1. A Larionov, A Krause, W Miller: **A standard curve based method for relative real time PCR data processing**. *BMC Bioinformatics* 2005, **6**:62.
