# Supplementary material for: Statistical significance of quantitative PCR
Source: BMC Bioinformatics. 2007 Apr 20;8:131. doi: 10.1186/1471-2105-8-131 (PMC1868764; doi:10.1186/1471-2105-8-131)
Supplement: Additional file 4 — Mathematical Justification of LinReg. Justification of the LinReg method to estimate PCR efficiency, when PCR is considered as a branching process. [file 1471-2105-8-131-S4.pdf]

Statistical significance of quantitative PCR: Additional File 4

Yann Karlen[1], Alan McNair[1], Sébastien Perseguer[2], Christian Mazza[3] and Nicolas Mermod[1]*

Mathematical justification of the Linreg method used to estimate PCR efficiency

The PCR can be modelled, under various simplifying assumptions, by a branching process [1-6], which can be described as follows: Let Z_c denote the number of molecules after c cycles of PCR. For each of the Z_c molecules, consider a binary variable ε_i which takes the value 1 with probability *p* and 0 with probability *1-p*, written as $P(\varepsilon_i = 1) = p$; $\varepsilon_i = 1$ meaning that molecule i duplicates during the (*c+1*)th cycle. Then the number of molecules at cycle c+1 is then given by

Eq. 1

$$Z_{c+1} = Z_c + \sum_{1}^{Z_c} \varepsilon_i$$

Where $\sum_{1}^{Z_c} \varepsilon_i$ is the number of duplicated molecules. The law of large numbers implicates that for large Z_c,

Eq. 2

$$\frac{1}{Z_c} \sum_{1}^{Z_c} \varepsilon_i \approx p$$

Let N_c denote the average value of Z_c. Then $N_c = Z_0 (1 + p)^c = Z_0 \cdot E^c$, $E = 1 + p$ in accordance with Eq.1 of the manuscript.

The exponential growth model describes thus the evolution of the averages values of the branching process. Z_c is in fact measured indirectly through fluorescence. Let us denote by $F_c = \alpha \cdot Z_c + error$ the measure of the fluorescence associated with Z_c, where α is a constant of proportion. As explained in the results, *Ct* values correspond to the point at which amplification curve crosses a fluorescence threshold which yields indirectly to a number Θ of molecules. Θ can be assumed to be a very large number, since a starting number of 10^5 molecules needs around 20 cycles to reach the threshold [7]. Let *Ct* be defined as the first cycle number c so that $Z_c \geq \Theta$. The data is censored to the subset $\left[Z_{Ct - M/2}, ..., Z_{Ct}, ..., Z_{Ct + M/2} \right]$, where M is the length of the observation period which is centered around *Ct*.

From Eq. 2 we can define the difference between the empirical frequency of duplication and the probability p of duplication as $y_{Ct+1} = \frac{1}{Z_{Ct}} \sum_{1}^{Z_{Ct}} \varepsilon_i - p$.

The central limit theorem implies that for large Θ, we can assume that y_{Ct+1} is approximatively a centered Gaussian of small variance $p(1-p)/Z_{Ct}$ (since Z_{Ct} is large). Therefore Eq. 1 becomes

Eq. 3

$$Z_{Ct+1} = Z_{Ct}\left(E + y_{Ct+1}\right)$$

Taking logarithms on both sides, and using a Taylor expansions as the variance is small,

$$\log(Z_{Ct+1}) = \log(Z_{Ct}) + \log(E) + y_{Ct+1}/E + 0(y_{Ct+1}^2)$$

Concerning the measurements, the fluorescence becomes

$$\log(F_{Ct+1}) = \log(F_{Ct}) + \log(E) + y_{Ct+1}/E + \Delta_{Ct+1}$$

where Δ_{Ct+1} is the error coming from fluorescence measurement, which is assumed to be a centered Gaussian of variance σ^2.

Proceeding similarly for the next cycles, one obtains the linear model

Eq. 4

$$\log(F_{Ct-M/2+k}) = \log(F_{Ct-M/2}) + k\log(E) + \left(y_{Ct-M/2} + ... + y_{Ct-M/2+k}\right)/E + \left(\Delta_{Ct-M/2} + ... + \Delta_{Ct-M/2+k}\right)$$

where $0 \le k \le M$ and all the errors y_i, Δ_k may be assumed to be independent. This is a linear model with correlated errors, where the correlation is created by the y-terms.
The covariance matrix D of the error process is given by

$$D_{kl} = \frac{p(1-p)}{E^2 \cdot Z_{Ct-M/2}} \cdot \min(k,l) + \sigma^2 \cdot \delta_{kl}$$

where $\delta_{kl} = 0$ when k is different from l, and equal to one otherwise. $Z_{Ct} > \Theta >> 1$ implies that the matrix can be approximated by $\sigma^2 Id$, where Id is the identity matrix. This also shows that the simple linear model with independent centered errors

$$\log(F_{Ct-M/2+k}) = \log(F_{Ct-M/2}) + k\log(E) + \Delta_k$$

is appropriated for large Θ, justifying in this way the use of the algorithms provided by LinReg.

Bibliography

1. S Schnell, C Mendoza: **Enzymological considerations for a theoretical description of the quantitative competitive polymerase chain reaction (QC-PCR)**. *J Theor Biol* 1997, **184**:433-40.
2. S Schnell, C Mendoza: **Theoretical description of the polymerase chain reaction**. *J Theor Biol* 1997, **188**:313-8.
3. F Sun: **The polymerase chain reaction and branching processes**. *J Comput Biol* 1995, **2**:63-86.
4. MV Velikanov, R Kapral: **Polymerase chain reaction: a Markov process approach**. *J Theor Biol* 1999, **201**:239-49.
5. P Jagers, F Klebaner: **Random variation and concentration effects in PCR**. *J Theor Biol* 2003, **224**:299-304.
6. G Stolovitzky, G Cecchi: **Efficiency of DNA replication in the polymerase chain reaction**. *Proc Natl Acad Sci U S A* 1996, **93**:12947-52.
7. J Wilhelm, A Pingoud, M Hahn: **Validation of an algorithm for automatic quantification of nucleic acid copy numbers by real-time polymerase chain reaction**. *Anal Biochem* 2003, **317**:218-25.
