# Supplementary material for: Statistical significance of quantitative PCR
Source: BMC Bioinformatics. 2007 Apr 20;8:131. doi: 10.1186/1471-2105-8-131 (PMC1868764; doi:10.1186/1471-2105-8-131)
Supplement: Additional file 5 — ΔCt systematic bias. When not fulfilled, the ΔCt assumption of equal efficiency induces a bias in induction estimates. Equations are developed to estimate the bias as a function of the real efficiency. [file 1471-2105-8-131-S5.pdf]

Statistical significance of quantitative PCR: Additional File 5

Yann Karlen[1], Alan McNair[1], Sébastien Perseguer[2], Christian Mazza[3] and Nicolas Mermod[1]*

[1]Institute of Biotechnology, University of Lausanne, 1015 Lausanne, Switzerland. [2]Physics department, Ecole Polytechnique Fédérale de Lausanne, 1015 Lausanne, Switzerland. [3]Section de Mathématique, University of Geneva, 2-4 Rue du Lièvre, 1211 Genève 24

ΔCt systematic bias

The ΔCt method was the first reported data processing strategy [1, 2]. It assumes that differences in the efficiency value of PCR reactions are negligible during the exponential phase, so that all efficiencies are estimated to take a value of 2 during this phase. Under this assumption,

Eq. 2

$$R_{AB} = \frac{A_0}{B_0} = \frac{E_B^{Ct_B}}{E_A^{Ct_A}}$$

can be rewritten

Eq. 2.1

$$R_{AB} = \frac{A_0}{B_0} = \frac{2^{Ct_B}}{2^{Ct_A}} = 2^{Ct_B - Ct_A} = 2^{\Delta Ct}$$

This assumption induces a theoretical bias that can be evaluated as follows. Considering an experimental setting with a sample diluted α-fold. Two PCR reactions are performed on both undiluted and α-diluted sample under the same experimental conditions (ie with the same efficiency deviation from the value of 2). The relationship between the original copy number of the undiluted tube (B) and the

α-diluted tube (A) is: $B_0 = \alpha \cdot A_0$. Expressing their ratio at the *Ct* threshold cycle (Eq.2) and rearranging leads to:

$$\alpha = \frac{B_0}{A_0} = \frac{E^{Ct_A}}{E^{Ct_B}} \Rightarrow E^{Ct_B} = \frac{1}{\alpha} \cdot E^{Ct_A}$$

where *E* is the true efficiency of both PCR reaction. This equation can be log-linearized:

$$Ct_B \cdot \log E = Ct_A \cdot \log E - \log \alpha \Rightarrow Ct_B = Ct_A - \frac{\log \alpha}{\log E}$$

Under the ΔCt assumption of all efficiencies equal to 2, the measured ratio R_{AB} (Eq. 2.1) becomes

Eq. 2.2

$$R_{AB} = 2^{Ct_A - Ct_B} = 2^{\log a / \log E}$$

Which is of course equal to α if E is equal to 2. Then the systematic error induced by this assumption (bias) can be expressed as the relative difference between the measured ratio and the actual dilution α:

Eq. 2.3

$$Bias = \frac{R_{AB} - \alpha}{\alpha}$$

Which is null when *E* is equal to 2.

The analysis of efficiencies shows that the Δ*Ct* assumption is not valid. This can be further illustrated by representing the systematic bias induced by the Δ*Ct* method when comparing known dilutions of the same sample. One can see that for primers having a measured efficiency of 1.8, which is about the center of the distribution of efficiencies (Figure 3 of the manuscript), the initial DNA concentration will be underestimated by 30% for a 10-fold dilution and by almost 60% for a 100-fold dilution ratio. Although this simulation clearly shows that the Δ*Ct* method is unsuitable for precise quantification, it was kept as a reference for comparisons with other methods.

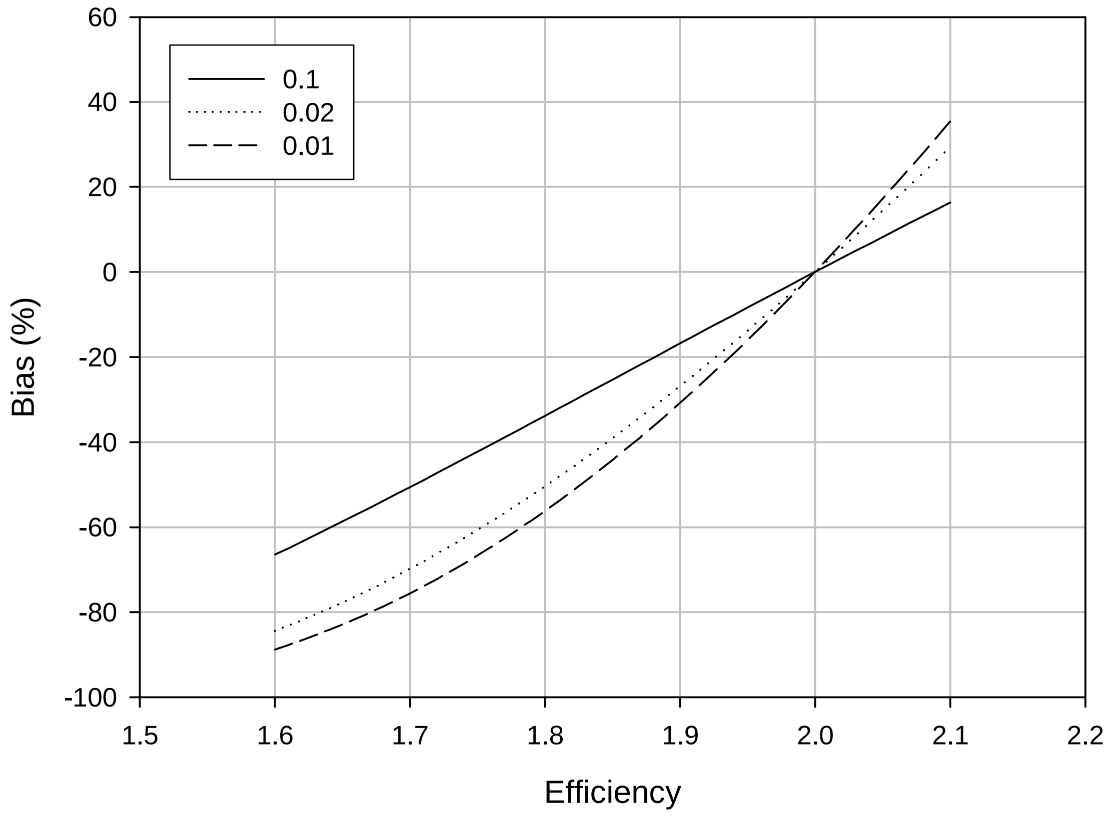

Simulation of the systematic measurement error (bias) induced by the ΔCt method.

The ΔCt method assumes that the efficiency equals 2, which induces an error on the calculation of the initial DNA concentration. This systematic error or bias can be quantified when comparing two samples of differing DNA concentration using the same primer set. The difference between the actual and the measured ratio (bias) is represented here as a function of the efficiency for two DNA concentrations that differ by a 10-fold, 50-fold or 100-fold molar ratio (0.1, 0.02 and 0.01, respectively).

Bibliography

1. KJ Livak, TD Schmittgen: **Analysis of relative gene expression data using real-time quantitative PCR and the 2(-Delta Delta C(T)) Method**. *Methods* 2001, **25**:402-8.
2. KJ Livak: **ABI Prism 7700 Sequence Detection System. User bulletin 2**. *PE Applied Biosystems* 1997.
