# Supplementary material for: Statistical significance of quantitative PCR
Source: BMC Bioinformatics. 2007 Apr 20;8:131. doi: 10.1186/1471-2105-8-131 (PMC1868764; doi:10.1186/1471-2105-8-131)
Supplement: Additional file 6 — Equation development. Detailed development of all equations 1–14 of the Methods section. [file 1471-2105-8-131-S6.pdf]

Statistical significance of quantitative PCR: Additional File 6

Yann Karlen[1], Alan McNair[1], Sébastien Perseguer[2], Christian Mazza[3] and Nicolas Mermod[1]*

Equations development

Gene expression normalization

PCR dynamics can be modelled by an exponential equation derived from the basic template DNA replication mechanism:

$$N_c = N_0 \cdot 2^c$$

Where N_c is the amount of PCR DNA product at cycle *c*. In a perfectly efficient PCR reaction, the amount, or the copy number of PCR DNA molecules, would double at each cycle but, due to a number of factors, this is rarely the case in experimental conditions. Therefore the equation above is generalized as:

Eq. 1

$$N_c = N_0 \cdot E^c$$

Where E is the PCR efficiency with $1 < E < 2$.

Because quantitative PCR measures fluorescence as an estimator of the amount of DNA present in the reaction at a given time, the absolute amount of initial DNA is generally unknown. Therefore the initial amount of cDNA corresponding to the gene of interest (DNA A) must be normalized to the level of an endogenous control gene or of a reference DNA standard (DNA B). Eq.1 can be formulated for the two DNA:

$$A_c = A_0 \cdot E_A^c$$
$$B_c = B_0 \cdot E_B^c$$

A_{Ct_A}=B_{Ct_B} when the amplification curves of each DNA cross the threshold fluorescence value, where Ct_A and Ct_B are the threshold cycles of the two genes. Thus the DNA concentration ratio becomes:

Eq. 1.2

$$\frac{A_{Ct_A}}{B_{Ct_B}} = \frac{A_0 \cdot E_A^{Ct_A}}{B_0 \cdot E_B^{Ct_B}} = 1$$

which resolves into:

Eq. 2

$$R_{AB} = \frac{A_0}{B_0} = \frac{E_B^{Ct_B}}{E_A^{Ct_A}}$$

where R_{AB} is the normalized ratio of the molar concentrations or of the copy number of DNA A relative to DNA B in a given sample. Note that Eq. is a simplified form of the one developed by Meijerink and colleagues [1].

Propagation of error on normalized expression

Assuming that in the second equality of Eq.2 E_A, Ct_A, E_B and Ct_B are obtained from imprecise measurements, the error on the normalized expression ratio may be determined using the Taylor expansion to the first order for the calculation of error propagation (or the delta method), which consists in expressing the derivative of R_{AB} as the sum of the partial derivative relative to each variable:

$$Var(R) = \sum_i \left(\frac{\partial R}{\partial x_i} \right)^2 \cdot Var(x_i)$$

where each x_i is a measured variables (E_A, Ct_A, E_B, Ct_B), and $Var(x_i)$ their associated measurement error.

The equation above is valid for large samples and is useful to determine mathematically the propagation of error. Due to the relatively small size of our data set, the continuous ∂x_i figures must be replaced by their discrete counterparts Δx_i to have a proper idea of how the errors propagates.

Eq. 11

$$\left|\Delta R_{AB}\right| \cong R_{AB} \cdot \sqrt{\left(\frac{Ct_A}{E_A}\right)^2 \cdot (\Delta E_A)^2 + (\ln E_A)^2 \cdot (\Delta Ct_A)^2 + \left(\frac{Ct_B}{E_B}\right)^2 \cdot (\Delta E_B)^2 + (\ln E_B)^2 \cdot (\Delta Ct_B)^2}$$

Note that this is an approximation of the behaviour of errors, whose approximated error tends to zero for large sample sizes, thus fulfilling the central limit theorem.

When considering the first equality of Eq.2 (ratio of the original amount of template DNA), ΔR_{AB} is given by:

Eq. 10

$$\left|\Delta R_{AB}\right| = R_{AB} \cdot \sqrt{\left(\frac{\Delta A_0}{A_0}\right)^2 + \left(\frac{\Delta B_0}{B_0}\right)^2}$$

Relative induction of gene expression

Usually, experimenters are interested in the difference of the gene expression between two conditions (with versus without a drug, sane tissue versus metastatic tissue, etc…) [2-4]. We want to know whether the expression of the gene of interest is induced or repressed upon treatment. So the useful figure is the ratio of the normalized ratios (Eq.2), that we will refer as the normalized induction ratio thereafter:

Eq. 13

$$I_{1-2} = \frac{R_{AB(1)}}{R_{AB(2)}}$$

where I_{1-2} is the difference of expression of the gene of interest (induction) between condition 1 and condition 2 and $R_{AB(i)}$ is the normalized expression of the gene interest in condition *i* (1 or 2). Note that Eq.13 is valid only if the gene used for the normalization (internal standard) has an expression that is invariant with condition 1 and 2 [5]. Error on induction values is given by

Eq. 14

$$\Delta I_{1-2} = I_{1-2} \cdot \sqrt{\left(\frac{\Delta R_{AB(1)}}{R_{AB(1)}}\right)^2 + \left(\frac{\Delta R_{AB(2)}}{R_{AB(2)}}\right)^2}$$

Estimation of the PCR efficiency

Determination of PCR efficiency can be readily performed by log-linearizing Eq.1:

Eq. 3

$$\log N_c = \log N_0 + c \cdot \log E$$

Eq.3 is a linear function of $\log N_c = f(c)$ with a slope $m = \log E$ when amplification is in the exponential phase. Therefore efficiency can be determined along with N_0 by performing a linear regression through the linear part of the amplification plot when data are represented on a half log scale (LinReg method) [6-8]. The intercept of this regression is directly linked to the original DNA molecule number in the reaction, and the initial ratio of two DNA molecules can be related to their ratio during exponential amplification as in the first equality of Eq. .

When $c = Ct$, Eq.3 can be rearranged as:

Eq. 4

$$Ct = -\frac{1}{\log E} \cdot \log N_0 + \frac{\log N_{Ct}}{\log E}$$

This expresses Ct as a linear function of $(\log N_0)$, with slope

Eq. 5

$$m = -\frac{1}{\log E} \Rightarrow E = 10^{-1/m}$$

Therefore, measuring *Ct*s of a set of serially diluted samples and performing a linear regression through a graph of the log of known initial (or relative) concentration vs their corresponding *Ct* allows the measurement of the efficiency [9]. This set of equations holds true only under the assumption that the PCR amplification behaves exponentially at the measured *Ct* and that the efficiency is identical between the different PCR amplifications.

Any linear regression generates an error on the fitted value of the slope and the intercept (if $r^2<1$). The regression function of Microsoft Excel was used to evaluate this error, providing the standard deviation of the slope and then calculating the associated error on the efficiency using the previous formulas for the propagation of errors:

Eq. 5.1

$$|\partial E| = \sqrt{(E \cdot \ln 10 \cdot \partial m)^2}$$

Propagation of error on averaged values

The different data processing models were first compared using experimental data averaged from all the conditions tested (primer, sample, dilution, etc), so as to avoid the potential bias that may result from the use of a particular primer or sample. Since each value used in the average has an associated error, we used a Taylor limited expansion method to determine how these errors propagate in the average value:

$$\overline{X} = \frac{1}{n}\sum_{i=1}^{n} x_i$$

with the expression for the error $\Delta\overline{X}$ determined by

$$|\Delta\overline{X}| = \frac{1}{n}\sqrt{\sum_{i=1}^{n} (\Delta x_i)^2}$$

References

1. J Meijerink, C Mandigers, L van de Locht, E Tonnissen, F Goodsaid, J Raemaekers: **A novel method to compensate for different amplification efficiencies between patient DNA samples in quantitative real-time PCR**. *J Mol Diagn* 2001, **3**:55-61.
2. R Pankiewicz, Y Karlen, MO Imhof, N Mermod: **Reversal of the silencing of tetracycline-controlled genes requires the coordinate action of distinctly acting transcription factors**. *J Gene Med* 2005, **7**:117-32.
3. DG Ginzinger: **Gene quantification using real-time quantitative PCR: an emerging technology hits the mainstream**. *Exp Hematol* 2002, **30**:503-12.
4. IM Mackay, KE Arden, A Nitsche: **Real-time PCR in virology**. *Nucleic Acids Res* 2002, **30**:1292-305.
5. SR Sturzenbaum, P Kille: **Control genes in quantitative molecular biological techniques: the variability of invariance**. *Comp Biochem Physiol B Biochem Mol Biol* 2001, **130**:281-9.
6. C Ramakers, JM Ruijter, RH Deprez, AF Moorman: **Assumption-free analysis of quantitative real-time polymerase chain reaction (PCR) data**. *Neurosci Lett* 2003, **339**:62-6.
7. A Gentle, F Anastasopoulos, NA McBrien: **High-resolution semi-quantitative real-time PCR without the use of a standard curve**. *Biotechniques* 2001, **31**:502, 504-6, 508.
8. JH Marino, P Cook, KS Miller: **Accurate and statistically verified quantification of relative mRNA abundances using SYBR Green I and real-time RT-PCR**. *J Immunol Methods* 2003, **283**:291-306.

9. KJ Livak: **ABI Prism 7700 Sequence Detection System. User bulletin 2**. *PE Applied Biosystems* 1997.
