# Supplementary material for: Statistical significance of quantitative PCR
Source: BMC Bioinformatics. 2007 Apr 20;8:131. doi: 10.1186/1471-2105-8-131 (PMC1868764; doi:10.1186/1471-2105-8-131)
Supplement: Additional file 7 — Statistical significance and required sample size. Presentation of all of the equations leading to the development of eq.15 of the Methods section. [file 1471-2105-8-131-S7.pdf]

Statistical significance of quantitative PCR: Additional File 7

Yann Karlen[1], Alan McNair[1], Sébastien Perseguer[2], Christian Mazza[3] and Nicolas Mermod[1]*

Statistical significance and required sample size

The general method to calculate the required numbers of replicates, knowing the statistical properties of a measurement procedure can be derived from a simple requirement. If I_{real} is the true value of the normalized induction ratio as defined by eq.13 in the main body text (which is unknown), I_{meas} is the measured normalized induction ratio (an estimator of the true value) and σ_I^2 is the variance of I_{meas}, then I_{real} must belong to an interval centered around I_{meas} with a certain degree of significance:

$$I_{real} \in \left[I_{meas} \pm {\sigma_I}/{\sqrt{n}} \cdot z_{1-\alpha/2} \right]$$

Which can be rewritten as:

Eq. 1

$$I_{real} = I_{meas} \pm {\sigma_I}/{\sqrt{n}} \cdot z_{1-\alpha/2}$$

Where n is the number of independent replicates and $z_{1-\alpha/2}$ is the normalized reduced value related to significance level α of the statistical test. Eq. 1 can be rewritten so to have *n* as a function of the other parameters:

Eq. 2

$$n = \frac{\sigma_I^2 \left(z_{1-\alpha/2} \right)^2}{\left(I_{real} - I_{meas} \right)^2}$$

If the expression of a gene is really induced *i*-fold (with *i* being unknown), the measured induction ratio will distribute around *i*, depending on the dataset size and statistical distribution. To conclude with a sufficient degree of certainty that gene expression is induced *i*-fold, we may arbitrarily set here that the measured (estimated) value should lie within a domain of confidence (Range) of 10 or 20% of the real *i* value (Range = 0.1 or 0.2). Thus, the size of the data set required to achieve this conclusion should be defined such that Eq. 2 is verified:

Eq. 3

$$I_{real} = I_{meas} \pm Range \cdot I_{meas}$$

Finally the standard deviation an induction (σ_I) is by definition related to the coefficient of variation (CV):

Eq. 4

$$CV = \frac{\sigma_I}{I} \Rightarrow \sigma_I = CV \cdot I$$

Therefore, Eq. 2 can be re-written:

Eq. 5

$$n = \frac{(CV \cdot I_{meas})^2 \left(z_{1-\alpha/2}\right)^2}{(Range \cdot I_{meas})^2} = \frac{CV^2 \left(z_{1-\alpha/2}\right)^2}{(Range)^2}$$

where the number of measurements that is needed to obtain a reliable estimation of induction is not dependent upon the induction ratio, but only on the CV, the range of confidence and the significance level.
